# Distinctive features of blood- and ascitic fluid-derived extracellular vesicles in ovarian cancer patients

**DOI:** 10.1186/s10020-025-01177-7

**Published:** 2025-04-21

**Authors:** Francesca Gorini, Camelia Alexandra Coada, Sarah Monesmith, Antonio De Leo, Dario de Biase, Giulia Dondi, Stella Di Costanzo, Francesco Mezzapesa, Ivan Vannini, Mattia Melloni, Sara Bandini, Flora Guerra, Riccardo Di Corato, Pierandrea De Iaco, Patrizia Hrelia, Anna Myriam Perrone, Sabrina Angelini, Gloria Ravegnini

**Affiliations:** 1 Department of Pharmacy and Biotechnology (FABIT), University of Bologna, Via Irnerio 48, 40126 Bologna, Italy; 2 Department of Medical and Surgical Sciences (DIMEC), University of Bologna, 40138 Bologna, Italy; 3 Solid Tumor Molecular Pathology Laboratory, IRCCS Azienda Ospedaliero-Universitaria di Bologna/Azienda USL di Bologna, Bologna, Italy; 4 Division of Oncologic Gynecology, IRCCS Azienda Ospedaliero-Universitaria di Bologna, Bologna, Italy; 5 IRCCS Istituto Romagnolo per lo Studio dei Tumori (IRST) “Dino Amadori”, Meldola, Italy; 6 Department of Biological and Environmental Sciences and Technologies, Università del Salento, Lecce, Italy; 7 Institute for Microelectronics and Microsystems (IMM), CNR, Lecce, Italy; 8 Center for Biomolecular Nanotechnologies, Istituto Italiano di Tecnologia, Arnesano, Italy; 9 Clinical Pharmacology Unit, IRCCS Azienda Ospedaliero-Universitaria di Bologna, Bologna, Italy; 10 Department of Morpho-Functional Sciences, University of Medicine and Pharmacy “Iuliu Hațieganu”, Strada Victor Babeş 8, 400347, Cluj-Napoca, Romania

**Keywords:** Ovarian cancer, Extracellular vesicles, Metastasis, Metastatic spread, miRNA

## Abstract

**Background:**

Ovarian cancer (OC) is a highly aggressive malignancy characterized by early dissemination of cancer cells from the surface of the ovary to the peritoneum. To gain a deeper understanding of the mechanisms associated with this intraperitoneal spread, we aimed to characterize the role of extracellular vesicles (EVs) in metastatic colonization in OC.

**Methods:**

To this purpose, a total of 150 samples of ascitic fluids, blood serum, tumor and normal tissues from 60 OC patients, were extensively analyzed to characterize the EVs released in blood and ascitic fluids of OC patients, in terms of size, expression of superficial epitopes and abundance of miRNAs biocargo.

**Results:**

A statistically significant difference in the size of EVs derived from ascitic fluid and serum was identified. Analysis of surface protein expression highlighted twenty epitopes with a significant difference between the two biological matrices, of which 18 were over- and two were under-expressed in ascitic fluid. With regard to miRNA levels, Principal Component Analysis (PCA) assessed four distinct clusters representing tumor tissue, normal tissue, ascitic fluid, and serum. A prominent difference in circulating miRNAs was observed in serum and ascitic fluid highlighting 98 miRNAs significantly deregulated (*P-adj* < 0.05) between the two bodily fluids. Deregulated miRNAs and epitopes underline an enrichment in ascites in components contributing to the metastatic spread.

**Conclusion:**

The results highlight a clear difference between the two biological fluids, suggesting that tumor selectively releases specific EVs populations in serum or ascites. In this context, it seems that ascites-derived EVs play a major role in modulating EMT and metastatic cascade, which is a key feature of OC.

**Supplementary Information:**

The online version contains supplementary material available at 10.1186/s10020-025-01177-7.

## Introduction

High-grade serous ovarian carcinoma (HGSOC) represents the most common histological type of ovarian cancer (OC). It is a highly aggressive malignancy characterized by early dissemination of cancer cells from the surface of the ovary to the peritoneum. This transcoelomic spread can lead to the formation of peritoneal metastases, which is a common characteristic of advanced OC and occurs in 70% of cases at the time of diagnosis (Micek et al. 2023). Although it initially displays good chemosensitivity, the development of resistance mechanisms influenced by pharmacokinetics as well as tumor and microenvironment factors results in a gradual decline in platinum sensitivity; this phenomenon significantly contributes to its elevated lethality (Zhang et al. 2022; Morand et al. 2021). In this context, the accumulation of malignant ascitic fluid within the peritoneal cavity promotes the formation of peritoneal metastasis. Malignant ascitic fluid serves as a reservoir of a complex mixture of soluble factors including tumor cells, tumor cell spheroids, circulating tumor cells (CTCs) and a variety of host cells (Ford et al. 2020). These components produce and are influenced by a wide range of tumor-promoting soluble factors and extracellular vesicles (EVs), contributing to the microenvironment that supports the metastatic process. According to the seed-and-soil theory, EVs, specifically exosomes and microvesicles, play a pivotal role in metastatic dissemination. These EVs ("the seed") are released directly by the tumor mass in the bodily fluids and can reach distant cells ("the soil") (Becker et al. 2016). EVs transfer their biological cargo, predominantly consisting of various types of RNAs, including miRNAs, mRNAs, and tRNAs, to the recipient cells, thus preparing the microenvironment of future metastatic sites before the arrival of cancer cells, ultimately leading to the formation of a pre-metastatic niche (Jabalee et al. 2018). EVs are known to be released in human fluids such as blood or ascites and could be involved in peritoneal dissemination. Nevertheless, they are poorly characterized and gaining a deeper understanding of the mechanisms associated with the intraperitoneal spread may foster the development of more effective strategies for the early detection and treatment of the disease (Xu et al. 2016; Dai et al. 2024; Tian et al. 2022). In this context, it has been suggested that the biofluids close to a tumor may be further enriched in tumor-derived substances, and this could be the case of ascites that completely surrounds the tumor (Wang et al. 2022).

The aim of the present study was to characterize the EVs released in blood and ascitic fluids of OC patients in terms of size, expression of superficial epitopes, and levels of miRNAs biocargo.

## Material and methods

### Patients selection and sample collection

The study enrolled patients who underwent primary surgery for advanced OC at the Division of Oncologic Gynecology, IRCCS Azienda Ospedaliero-Universitaria of Bologna, Italy, from 2021 to 2023. This study was conducted in accordance with the principles of Helsinki Declaration. Informed consent was obtained from all participants; the study received approval by the Institutional Review Board (788/2021/Sper/AOUBo) and it was registered to the ClinicalTrials.gov (Identifier: NCT05146505). For our investigative analysis, we systematically collected tumor and normal tissue specimens, ascites, and blood serum samples from patients during surgery. Inclusion criteria were: (i) patients with histologically confirmed HGSOC, (ii) who had not received neoadjuvant chemotherapy and (iii) who had no history of other cancers. Exclusion criteria were: (i) patients with a history of previous radiotherapy or chemotherapy and (ii) the absence of ascites.

### Sample processing

Tumor and normal tissue samples were collected through surgical biopsy during surgery, obtained from both the primary tumor (ovary) and abdominal skin (healthy tissue). Tissue samples were processed according to established protocols to ensure consistency and reliability. In particular, the specimens removed from the patients were preserved in sterile cryovials and stored in dry ice to avoid any potential material degradation; samples were then stored at −80 °C until use. Ascites, obtained through abdominal aspiration preceding any surgical procedure, along with blood samples, were meticulously processed to extract biological information. In particular, for the sera collection, approximately 5 mL of whole blood were collected in BD Vacutainer^®^ SST^™^ Tubes and processed within 2 hours from the collection. Blood was centrifuged at 3000 rpm for 10′ at 4 °C; the resulting supernatant was transferred into new vials without disturbing the serum separator gel, and a second centrifugation at 14,000 rpm for 15′ was performed at 4 °C. The supernatant was then transferred to cryovials and stored at −80 °C until use. Ascitic fluid was collected in sterile containers; the samples were aliquoted in 15 mL tube and centrifuged at 2000 rpm for 10′. The supernatant was transferred to 1.5 mL vials without disturbing any pellet and centrifuged at 14,000 rpm for 15′. Supernatant was collected in cryovials and stored at −80 °C until use.

### EVs isolation from serum and ascites samples

For EVs isolation from serum and ascites samples, qEV10 Size Exclusion Columns (70 nm, Izon Science Christchurch, New Zealand) were used following the company's protocol. Briefly, after rinsing the columns with PBS 1X, 500 µL of samples were applied on the top of a qEV column, and fractions were collected. Three vesicle–enriched fractions (Xu et al. 2016; Dai et al. 2024; Tian et al. 2022) were pooled together and evaluated through Nanoparticle Tracking Analysis (NTA).

### Nanoparticle tracking analysis (NTA)

A total of 74 matched samples from 37 patients were analyzed through NTA. NTA was used to determine particle size and estimate number of isolated EVs per milliliter from patient samples. EVs were analyzed by NTA with a NanoSight NS300 instrument (Malvern, United Kingdom), equipped with NTA 2.3 analytical software laser. The software calculates the mean diameter, mode diameter (nm), and concentrations of EVs. Software settings for analysis were maintained for all measurements. Data were analyzed with the NTA version 2.3.

### Transmission electron microscopy (TEM)

EVs were also analyzed by Transmission Electron Microscopy (TEM) to confirm the size observed by the NTA. For sample preparation, a 5 µL drop of a concentrated vesicle suspension was dropped on a Formvar-coated copper grid and after 30', infiltrated with a carboxymethyl dextran solution. The resulting ultrathin polysaccharide layer prevents the vesicle collapse on the dried grids. No staining was applied in sample preparation or after deposition on a grid. TEM analysis was performed with a JEOL JEM-1011 transmission electron microscope at 100 kV operating voltage, equipped with a 7 megapixel CCD camera (Orius SC600A, Gatan, Pleasanton, CA). TEM image analysis was achieved with Gatan Digital Micrograph^™^ (DM) software.

### Bead-based multiplex exosome flow cytometry assay

Expression of specific surface markers was evaluated by flow cytometry through the MACSPlex Exosome Kit (Miltenyi Biotec, Bergisch-Gladbach, Germany). The kit allows the detection of 37 exosomal surface epitopes (CD3, CD4, CD19, CD8, HLA-DR, CD56, CD105, CD2, CD1c, CD25, CD49e, ROR1, CD209, CD9, SSEA4, HLA-BC, CD63, CD40, CD62P, CD11c, CD81, MCSP1, CD146, CD41b, CD42a, CD24, CD86, CD44, CD326, CD133/1, CD29, CD69, CD142, CD45, CD31, CD20, and CD14) and two additional isotypes as a control (REA and mIgG1). EV-containing samples were processed as previously described (Bandini et al. 2021). For each sample, the 39 bead populations were distinguished by different fluorescence intensities detected in the FITC, PE, and APC channels by flow cytometric analysis carried out through a BD FACSCanto equipped with two lasers, 488 nm and 630 nm (Becton Dickinson, San Diego, CA, USA). Final analysis was performed through the BD FACSDiva software (Becton Dickinson, San Diego, CA, USA); raw median fluorescence intensity (MFI) of each marker was normalized by using the MFI of the negative control included in the same experiment. Serum and ascites derived EVs were compared.

### RNA isolation

Total RNA was isolated from tumor and non-tumor frozen samples. In detail, a small portion of the tumor was homogenized with TRIzol^™^ Reagent (Invitrogen, Carlsbad, CA) by using a homogenizer and then RNA extraction was performed following the manufacturer’s protocol. RNA quality was then assessed through Agilent 2000 bioanalyzer and quantified by Qubit.

RNA from biofluids was isolated through the exoRNeasy Midi Kit (Qiagen), starting from 450 µL of serum or ascites, following the manufacturer’s instructions. The ath-159a miRNA spike-in was used to check the quality of the extraction.

### Analysis of miRNA levels

miRNA levels were evaluated in 60 samples from 15 patients (15 sera, 15 ascites, 15 tumor samples, and 15 normal tissues) by the TaqMan Low Density Array (TLDA) Advanced miRNA Human A Cards (Applied Biosystems Cat No, A34714) containing 384 miRNA probes. For each patient, the abundance of miRNAs was evaluated in serum and ascites; in addition, we evaluated it in the tumor and in normal tissue too. For the liquid samples, 2 μl of RNA eluate were reverse transcribed to cDNA, using the TaqMan Advanced miRNA cDNA synthesis kit (Applied Biosystems Cat No. A28007) following the manufacturer’s protocol. For tumor and non-tumor samples, 10 ng of total RNA was used as input to synthesize cDNA, by using the same kit. The cDNA was loaded into the TaqMan Advanced miRNA array Card A and run in a QuantStudio 7 Flex system (Applied Biosystems). Before proceeding with the miRNA profiling, miR-16-5p (ID: 477860_mir) and ath-159a (ID: 478411_mir) levels were analyzed to carefully inspect the amplification.

Array data were normalized using the standard internal reference miR-16-5p, selected as internal reference after assessment of its stability in our study cohort and further review of the literature (Rinnerthaler et al. 2016). Data were analyzed using the 2^−ΔΔCt^ method and miRNAs with Ct > 35 were considered as not expressed and excluded from further analysis. Moreover, AMP scores returned from the instrument were used as parameters of the goodness of the Real Time (RT) reactions.

Validations for nine of the most significant miRNAs were carried out in a cohort made up of 60 patients (120 matched samples), using RT-PCR in a single probe test: miR-181b-5p (ID: 478583), miR-200a-3p (ID: 478490), miR-200b-3p (ID: 477963), miR-200c-3p (ID: 478351), miR-21-5p (ID: 477975), miR-2110 (ID: 477971), miR-325 (ID: 478025), miR-429 (ID: 477849) and miR-455-5p (ID: 478113).

Data were analyzed using the Thermofisher Cloud App with a threshold set to 0.1.

### Bioinformatic analysis of miRNA profiles, miRNA target prediction and functional enrichment

Levels of miRNAs in EVs released in serum and ascites were compared. R-Bioconductor package *limma* was adopted to evaluate the differential abundance profile. An adjusted *P-value* < 0.05 was considered significant. A heatmap was plotted by ttp://www.bioinformatics.com.cn/srplo, an online platform for data analysis and visualization. ﻿Principal component analysis (﻿PCA) was performed using the R CRAN package *rgl *and visualized through Cubemaker online tool.

Target genes of the validated miRNAs were predicted by miRTarBase and TargetScan Human 8.0; shared predicted genes were used to generate an epigenetic network and Cytoscape was employed to visualize the results of the protein interaction network. Functional enrichment was done using Gene Oncology (GO) annotations using *clusterProfiler* and *enrichplot* R packages. Biological process (BP), molecular function (MF), and cellular components (CC) were used and clustered based on their parent–child hierarchy relationships.

### In vitro validation of miRNA target genes

SKOV-3 (HTB-77) and OVCAR-3 (HTB-161) OC cell lines were purchased from American Type Culture Collection, ATCC (Manassas, VA, USA). SKOV-3 cells were cultured in IMDM supplemented with 10% FBS; OVCAR-3 cell line was cultured in RPMI supplemented with 10% FBS. Both the cell lines were cultured at 37 °C in a humidified atmosphere containing 5% CO2, and media change was performed twice a week. Mycoplasma tests (MycoAlert Assay, Lonza Walkersville) were performed regularly on a 4-month basis.

OC cell lines were transfected with 5 nM of mirVana miRNA mimic for miR-200, miR-429, and miR-21 (Thermo Fisher Scientific) using Lipofectamine RNAiMAX (Thermo Fisher Scientific, #13778150) according to the manufacturer’s protocol. Cells were also treated with the delivery agent alone to control for cellular effects due to transfection reagent exposure (indicated as “mock”). To evaluate miRNA and mRNA expression, total RNA was isolated and reverse transcribed through TaqMan Advanced miRNA cDNA synthesis kit (Applied Biosystems) and High-Capacity RNA-to-cDNA Kit (Applied Biosystems), respectively, 48 hours after miRNA mimics transfection. MiRNA levels were evaluated using TaqMan assays as previously described using miR-16-5p as control; mRNA levels for *ZEB1*, *CDH1* (E-cadherin), *CDH2* (N-cadherin), and *PTEN* were assessed by PowerUp^™^ SYBR^™^ Green Master Mix (Thermo Fisher Scientific). Relative expression was estimated by the ΔΔCt method, using *GAPDH* as housekeeping control. RT-PCR experiments were run in triplicate.

### Statistical analysis

Statistical analysis was carried out using the GraphPad Prism 8 and R version 4.4.2. Descriptive statistics are provided for all analyses. Continuous variables were presented as median and interquartile range (IQR), while categorical variables were presented as absolute frequency and percentage. Differences between groups for continuous variables were tested using the t-test or Mann-Whitney test after evaluation of normal distribution. For the survival impact of each miRNA, the cohort of patients was divided into two groups based on the abundance of the miRNA of interest using the median ΔCt value as a threshold. Kaplan–Meier curves were plotted for the overall survival (OS) and progression free survival (PFS) while the statistical significance was assessed using the log-rank test. A *P-value* < than 0.05 was considered statistically significant.

## Results

Out of the initial 106 patients, 19 were excluded from the analysis as they lacked a diagnosis of HGSOC. Additionally, 21 patients were excluded due to the absence of ascites. Four patients were further excluded because their tumor tissue samples were limited for diagnostic procedures. Furthermore, two patients were excluded due to observed hemolysis during serum separation, which could potentially compromise the results of subsequent analyses. After applying the exclusion criteria, 60 patients were eligible for comprehensive analysis. Supplementary Figure S1 and Table S1 report the general workflow of the analysis and summarize the main clinical features of the included patients, respectively.

### NTA

**Fig. 1:**
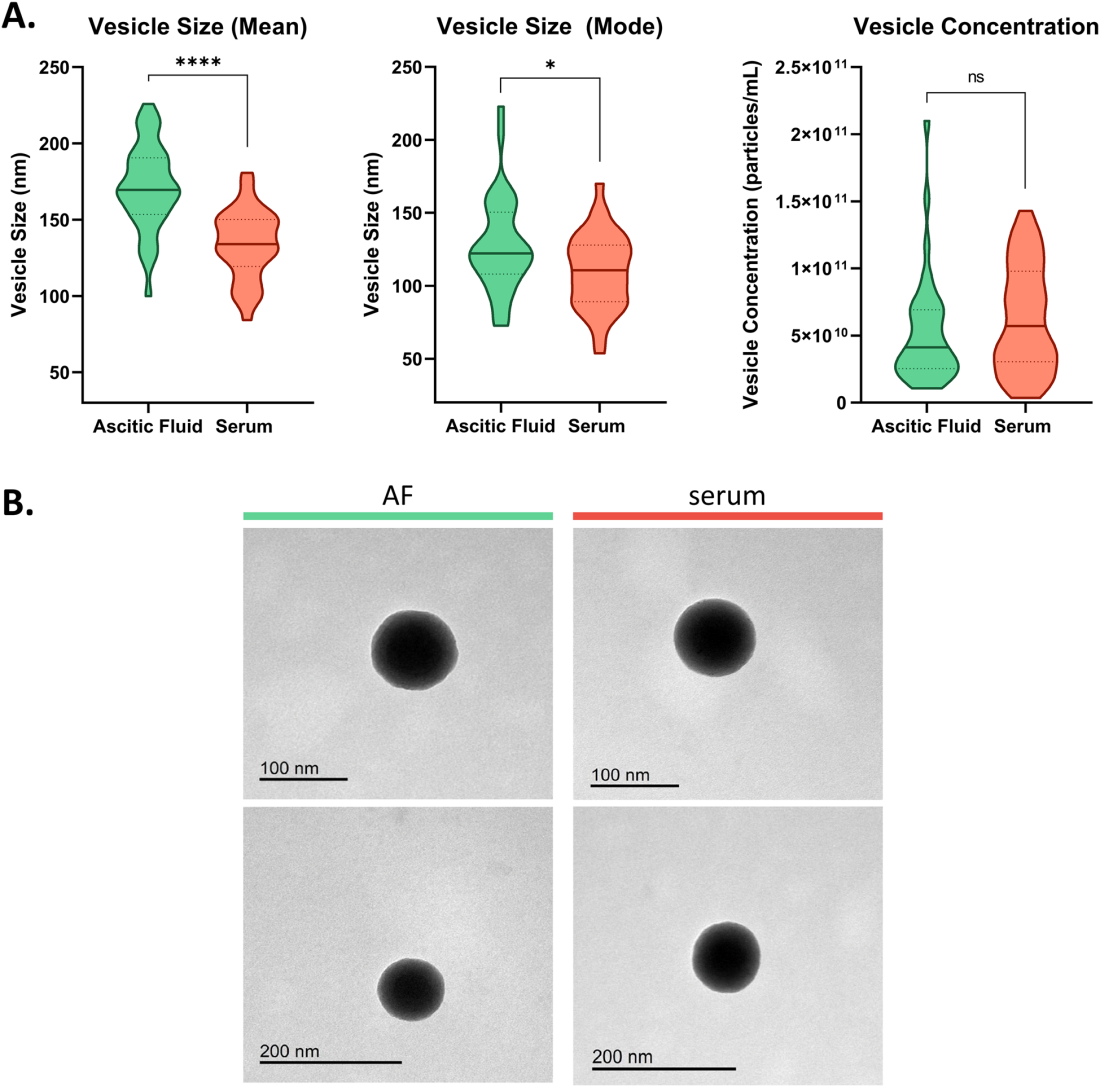
**A** Violin plots representing the vesicle size and concentration in serum and ascitic fluid samples, analyzed by NTA. Median and quartiles are represented by the bold line and dotted lines, respectively. **** = *P* < 0.0001; * = *P* < 0.05; *ns* not significant. **B** Representative images of EVs retrieved from blood serum (on the right) and ascitic fluid (AF, on the left) samples, analyzed by transmission electron microscopy (TEM)

The results highlighted a statistically significant difference in terms of size of the EVs derived from ascitic fluid and serum. In particular, mean size of EVs detected in serum was 136.4 ± 20.6 nM compared to a mean size of 171.6 ± 27.9 nM in ascitic fluid (*P* < 0.0001). Similarly, mean values of mode were respectively 113.2 ± 23.5 nM and 127.3 ± 32.4 nM (*P* = 0.04). With respect to the number of EVs per mL, we did not observe a statistically significant difference between the two bodily fluids even if a slightly higher concentration was retrieved in serum (serum vs ascites: 6.3E + 10 vs 5.2E + 10 particles/mL, *P* = 0.28). Results are shown in Fig. 1A.

### TEM

TEM analysis confirmed the size measured by the NTA. Examples of EVs observed in blood serum and ascites obtained by TEM are reported in Fig. 1B.

### Characterization of surface EV markers

**Fig. 2:**
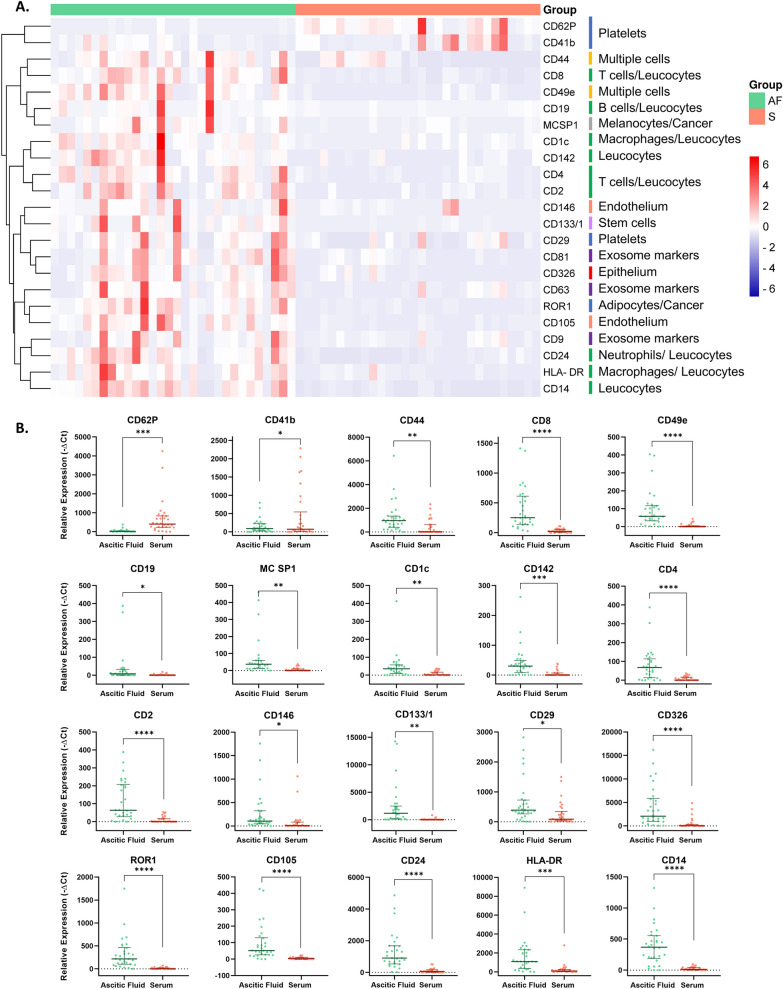
**A** Heatmap showing the expression of significant epitopes in serum (S) and ascitic fluid (AF). **B** Different expression of specific epitopes in EVs derived from ascitic fluid and serum. **** = *P* < 0.0001; *** = *P* < 0.001; ** = *P* < 0.01: * = *P* < 0.05

Analysis of surface protein expression was performed on the EVs in 60 samples (of which 30 serum samples and 30 ascites samples) using a protein multiplex bead-based flow cytometry assay as previously reported (Wiklander et al. 2018). MFI values of each marker expressed on EVs released in ascitic fluids and in serum were compared; Fig. 2A shows the heatmap of the epitope expression in the two bodily fluids. Expression of typical exosomal markers (CD9, CD63, CD81) was observed in both the bodily fluids even if in ascitic fluids MFIs were higher than in serum, with *P* = 0.0087, *P* = 0.0031, and *P* = 0.0002, respectively. Twenty epitopes showed a significant difference in the two matrices and, in particular, eighteen were over-expressed in ascitic fluid while two (CD62P and CD41b) were over-expressed in serum EVs. Table 1 summarizes the list of the differentially expressed epitopes in serum and ascitic fluid EVs.

**Table 1:** Differentially expressed epitopes in ascitic fluid and serum EVs

Epitope	*P-value*	MFI in AF	MFI in serum
CD4	< 0.0001	80.57	8.22
CD8	< 0.0001	398.43	31.85
CD105	< 0.0001	97.07	5.19
CD2	< 0.0001	114.40	12.44
CD49e	< 0.0001	95.57	5.56
ROR1	< 0.0001	326.07	12.74
CD24	< 0.0001	1308.33	104.48
CD326/EpCAM	< 0.0001	4041.70	601.33
CD14	< 0.0001	390.47	24.48
HLA-DR	0.0002	1660.30	273.30
CD62P	0.0003	43.97	701.82
CD142	0.0006	41.233	6.26
CD133/1	0.0012	2389.13	75.11
CD44	0.0033	1207.87	437.74
MCSP1	0.0040	77.70	5.96
CD1c	0.0065	48.03	9.56
CD29	0.0109	665.33	260.48
CD41b	0.0363	148.93	441.89
CD19	0.0377	37.80	2.07
CD146	0.039	271.17	97.00

*MFI:* raw median fluorescence intensity, *AF:* ascitic fluid

The topmost expressed epitopes in ascitic fluid EVs were CD326/EpCAM, CD133/1, HLA-DR, CD24 and CD44, which are cancer-related markers. However, the most expressed ones in serum EVs were CD62P and CD42a (platelet activation markers) and CD86 (an immunologic related protein) (Ekström et al. 2022). CD326 was also among the most expressed in serum EVs but not as high as in ascitic fluid (see Table 1 for the details). Figure 2B reports the difference in expression of EVs’ epitopes in serum and ascitic fluid.

### Analysis of miRNA levels

**Fig. 3:**
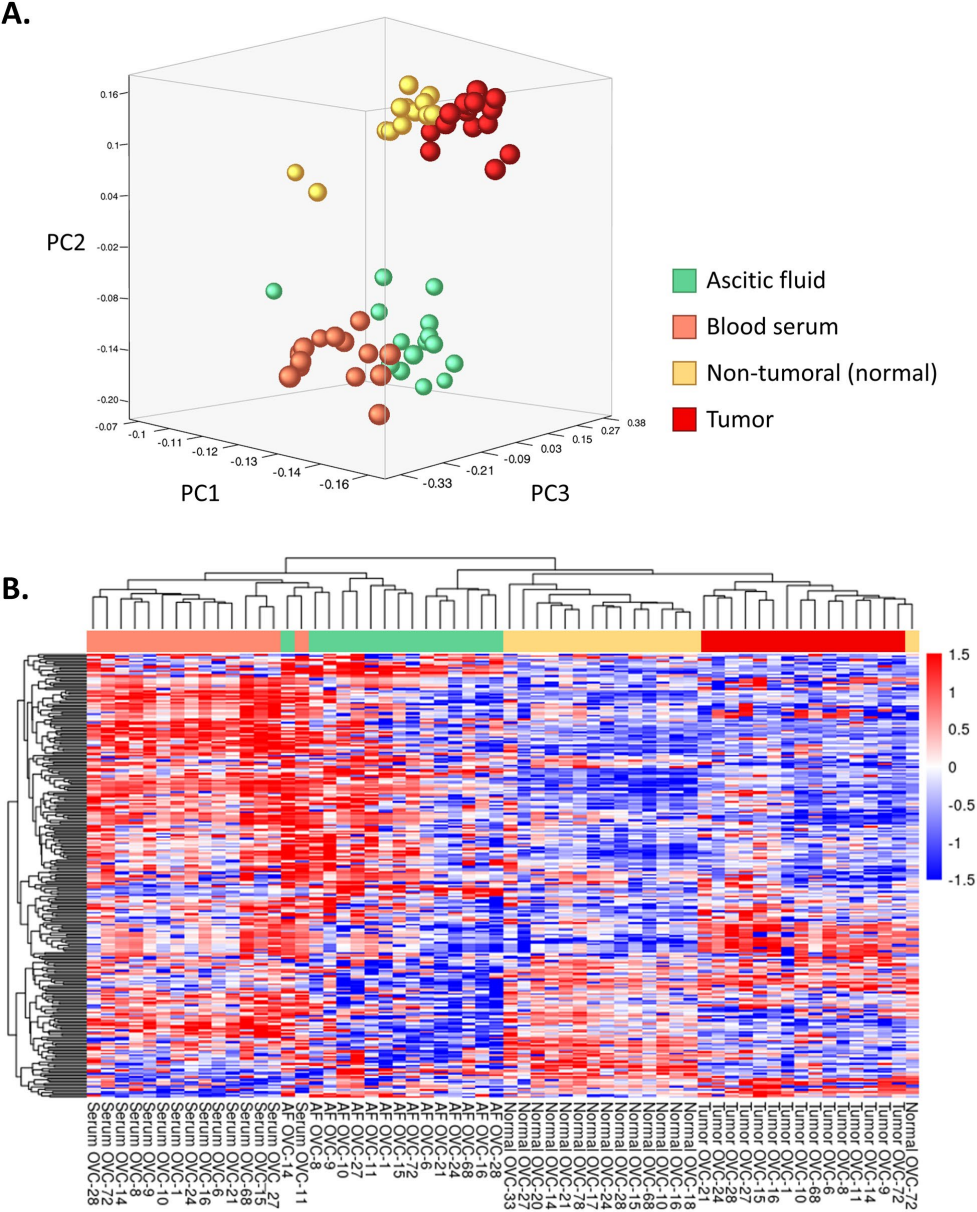
**A** PCA showing miRNAs levels in ascitic fluid, serum, tumor and non-tumor (normal) tissue samples. **B** Unsupervised Hierarchical clustering of miRNAs profile in blood serum, ascitic fluid, normal and tumor tissue of OC patients. Red and blue represent higher or lower levels of miRNAs (median centered), respectively. *AF:* ascitic fluid

PCA was performed as a means of quality control to assess the biological separation of the groups based on miRNA levels (Fig. 3A). Four distinct clusters were clearly identified, corresponding to tumor tissue, normal tissue, ascitic fluid, and serum. Notably, the figure highlights the distinct profiles of EV miRNAs in the two biofluids (serum and ascitic fluid) compared to those isolated from tumor and non-tumor tissues. We also applied the PCA to the tissue samples only, observing two separate clusters with no overlapping (Supplementary Figure S2).

The unsupervised heatmap representing miRNAs levels in the four matrices is shown in Fig. 3B. To identify the difference between miRNAs cargo in serum and ascites derived EVs, we compared the miRNA profiles. The analysis highlighted 98 miRNAs significantly deregulated (*P-adj* < 0.05) between the two bodily fluids. Considering the AMP score, 59 out of 98 miRNAs had AMP score > 1 in at least 50% of the samples; these miRNAs are reported in Table 2. Of the 59 significantly deregulated miRNAs, 11 were down-regulated in ascites, as indicated by the higher ΔCt value compared to serum, while 48 were up-regulated in ascitic fluid compared to serum.

**Table 2:**
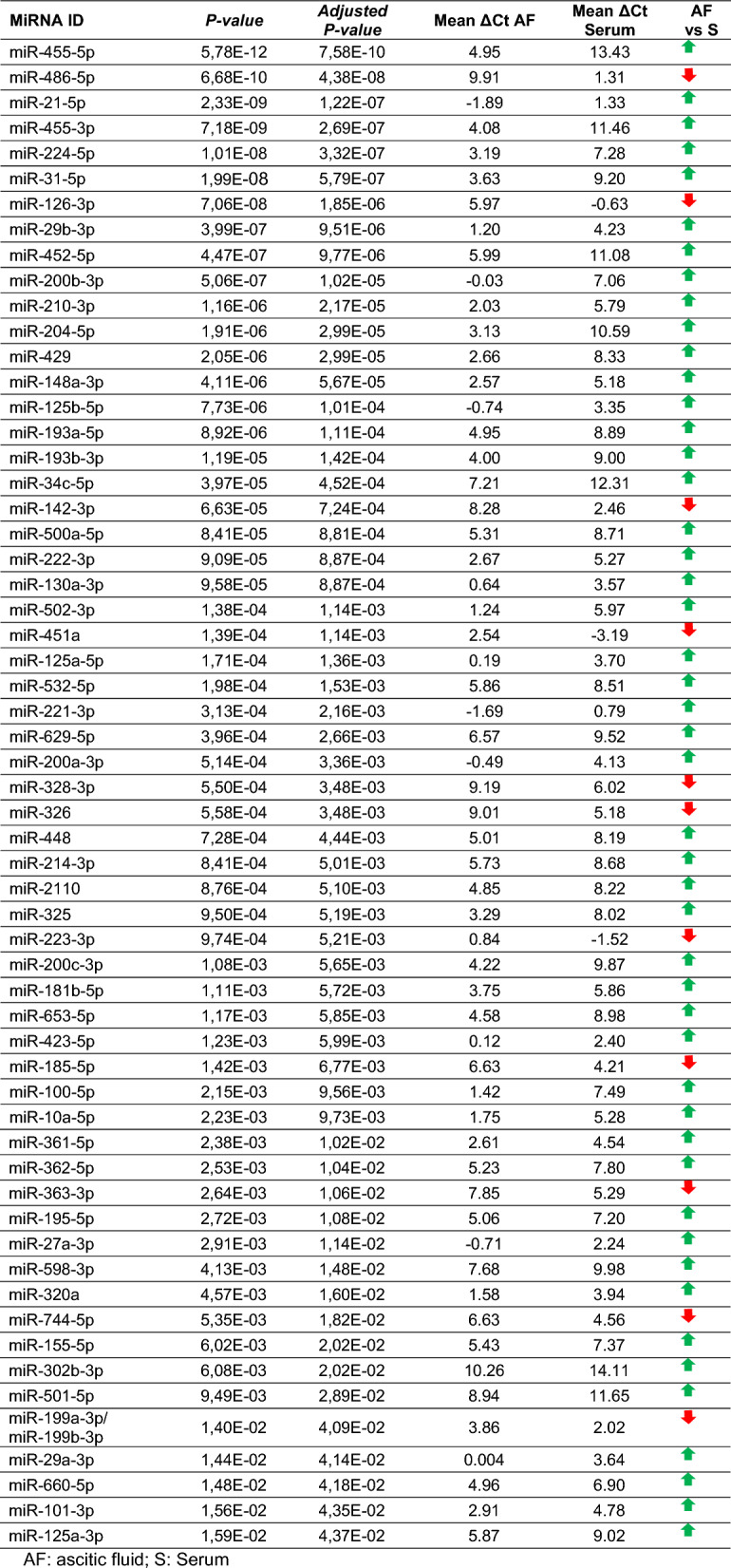
Significantly deregulated miRNAs between ascitic fluid and serum

*AF:* ascitic fluid, *S:* Serum

**Fig. 4:**
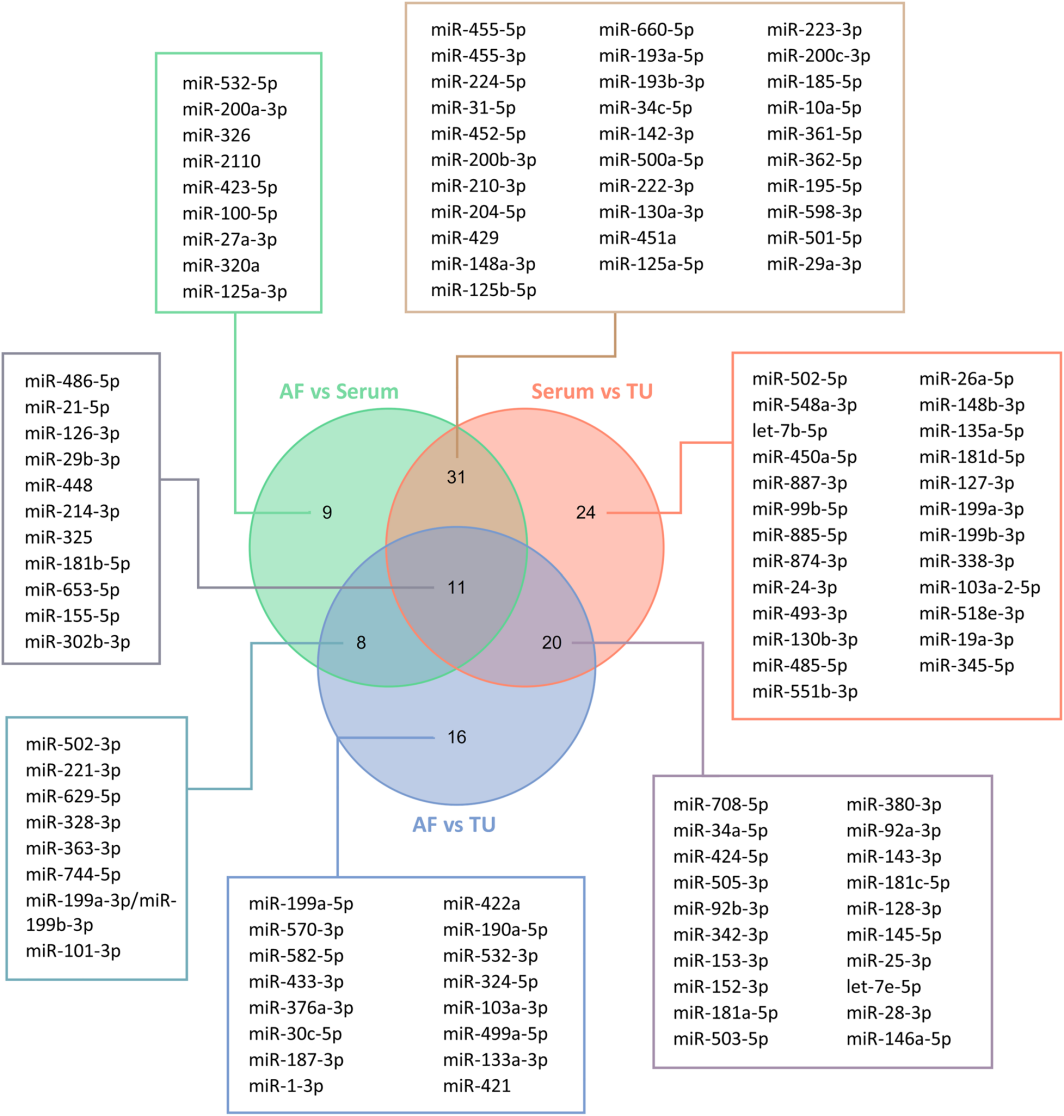
Venn diagram summarizing the results obtained from the different comparisons. *AF:* ascitic fluid, *TU:* tumor

Subsequently, profiles of miRNAs in serum and tumor tissue were compared: 143 miRNAs resulted significantly deregulated (*P-adj* < 0.05) between the two types of samples, of which 86 with an AMP score > 1 in at least 50% of the samples and are reported in Supplementary Table S2. Of these, 17 were up-regulated and 69 were down-regulated in serum with respect to tumor tissue. Moreover, profiles of circulating miRNAs in ascitic fluid and miRNAs in tumor tissue were compared: 113 miRNAs were significantly deregulated (*P-adj* < 0.05) between the two biological matrices, 55 of these with an AMP score > 1 in at least 50% of the samples and are reported in Supplementary Table S3. Of these, 16 were up-regulated and 39 were down-regulated in ascitic fluid with respect to tumor tissue. In total, 11 miRNAs resulted deregulated in all the comparisons (miR-486-5p, miR-21-5p, miR-126-3p, miR-29b-3p, miR-448, miR-214-3p, miR-325, miR-181b-5p, miR-653-5p, miR-155-5p, miR-302b-3p). Figure 4 summarizes the results deriving from these comparisons.

Finally, miRNA levels in normal tissues were compared with tumor tissue and reported in Supplementary Tables S4. In particular, 46 miRNAs resulted significantly deregulated (*P-adj* < 0.05) in normal compared with tumor tissue.

### Validation of the profiling results by qRT-PCR

Based on the statistical significance in the miRNA profiling and literature review, 9 miRNAs (miR-21-5p, miR-181b-5p, miR-200a-3p, miR-200b-3p, miR-200c-3p, miR-325, miR-429, miR-455-5p and miR-2110; all up-regulated in ascitic fluid vs serum) were selected for single miRNA assay validation in 60 patients, for a total of 120 matched samples.

**Fig. 5:**
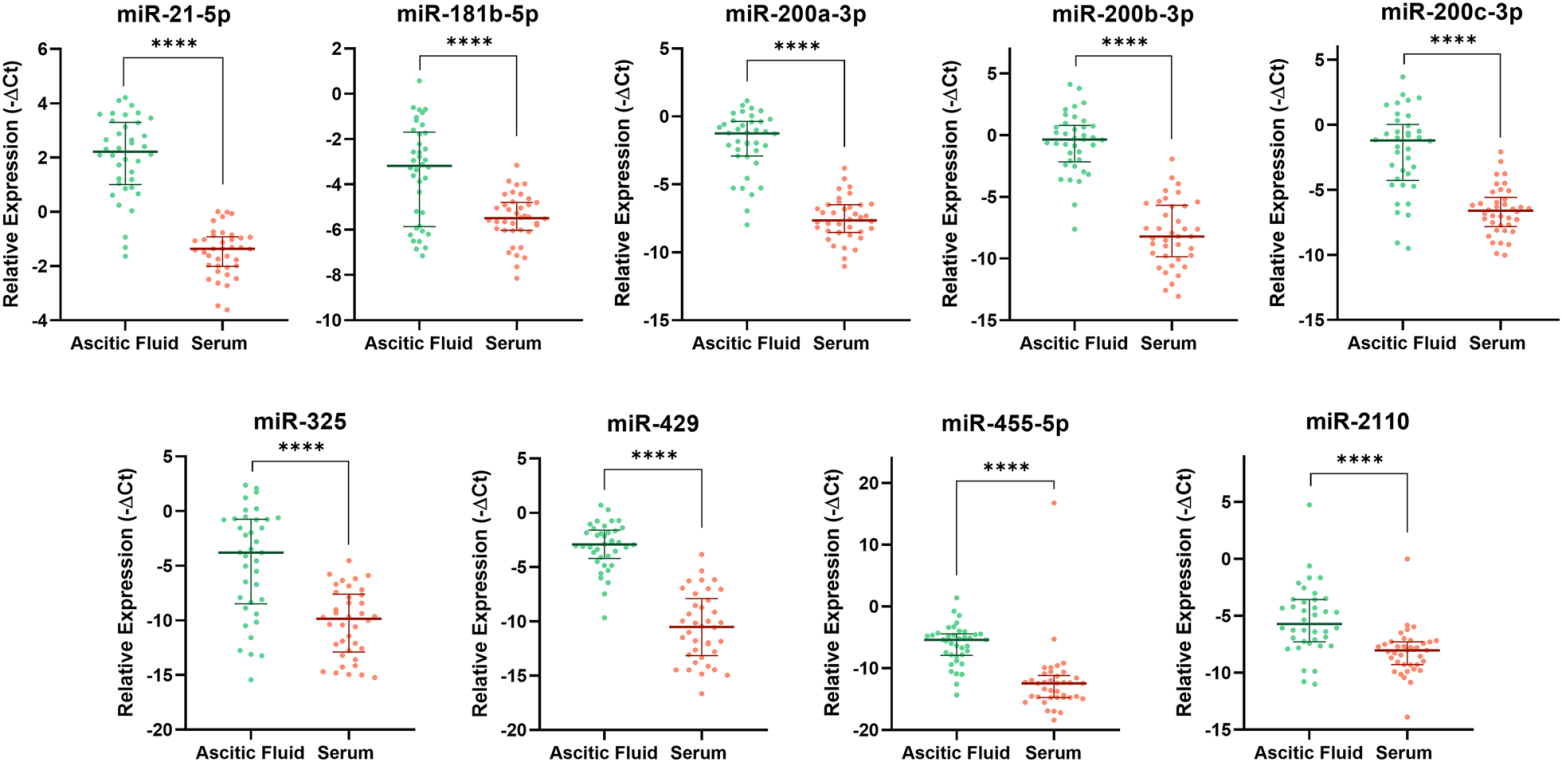
Relative abundance of the selected miRNAs. Median and interquartile ranges are presented. **** = *P* < 0.0001

Since our study was mainly focused on biological fluids, miRNA validation was performed in the sole ascitic fluid and serum. As shown in Fig. 5, all the selected miRNAs maintained their level of significance, being at higher levels in ascites (*P* < 0.0001), and confirmed the profiling results.

### Functional enrichment of miRNA gene targets

**Fig. 6:**
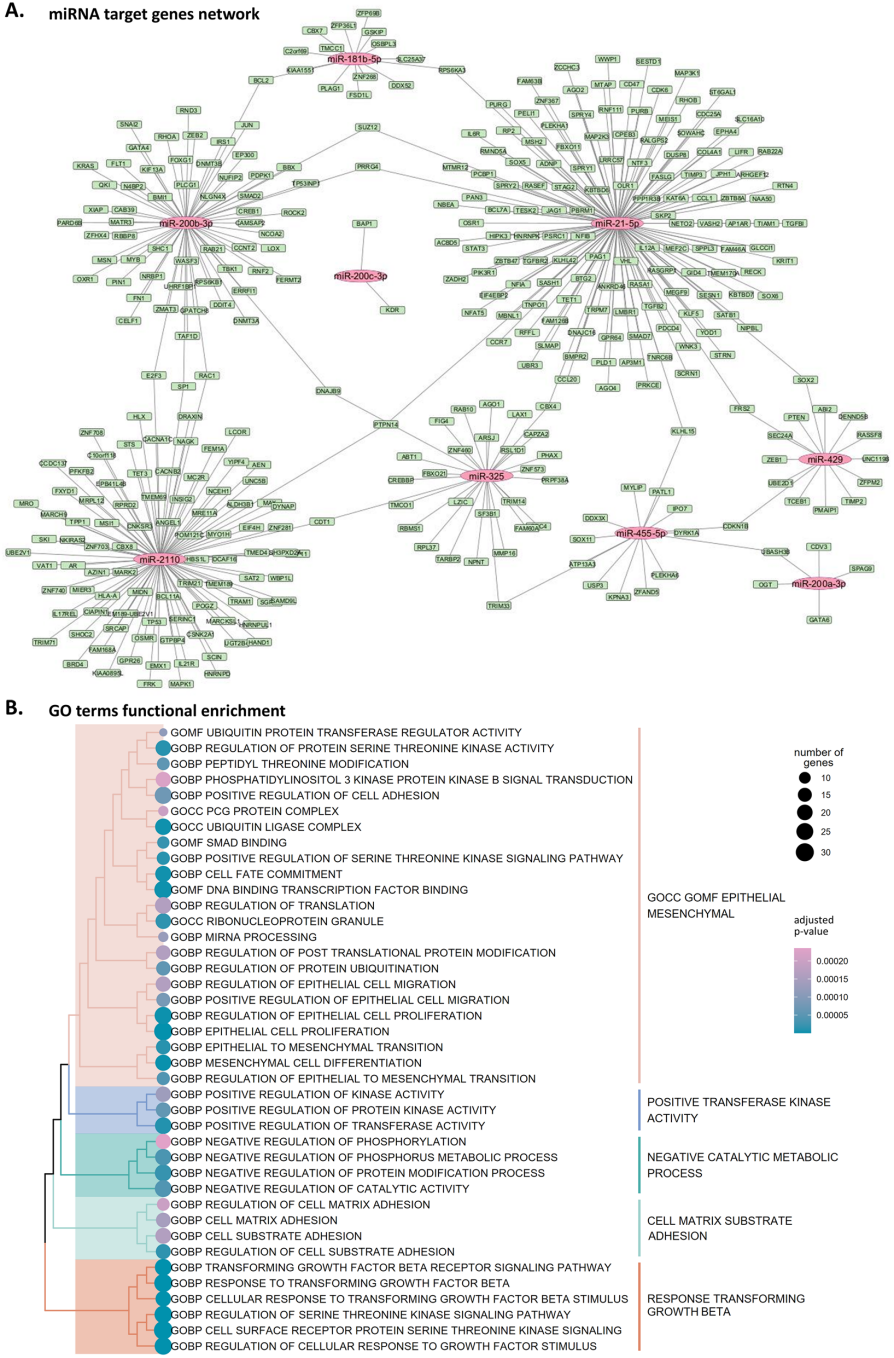
**A** Potential epigenetic networks between miRNAs and target genes. miRNAs are presented as central nodes colored pink, while their predicted target genes are shown in green. Some miRNAs have shared common targets. **B** Functional enrichment of Gene Ontology (GO) terms based on the significantly enriched miRNA target genes. The terms are divided into biological process (BP), molecular function (MF) and cellular compartment (CC). Size of dots indicates the number of genes involved in the specific pathways while their color indicates the adjusted *P-value.*

TargetScanHuman 8.0 and miRTarBase tools were used to predict the target genes for the selected miRNAs. The resulting predicted genes for each miRNA are reported in Supplementary Table S5. The miRNA-gene networks revealed multiple common gene targets suggesting a complex, multilayered regulation (Fig. 6A). Functional enrichment analysis using GO annotation revealed a significant enrichment of multiple terms related to key processes in cancer metastasis, including cell surface adhesion, epithelial-to-mesenchymal transition (EMT), protein synthesis, and response to growth factors signaling. The most significant results are presented in Fig. 6B.

### In vitro validation of miRNA target genes

**Fig. 7:**
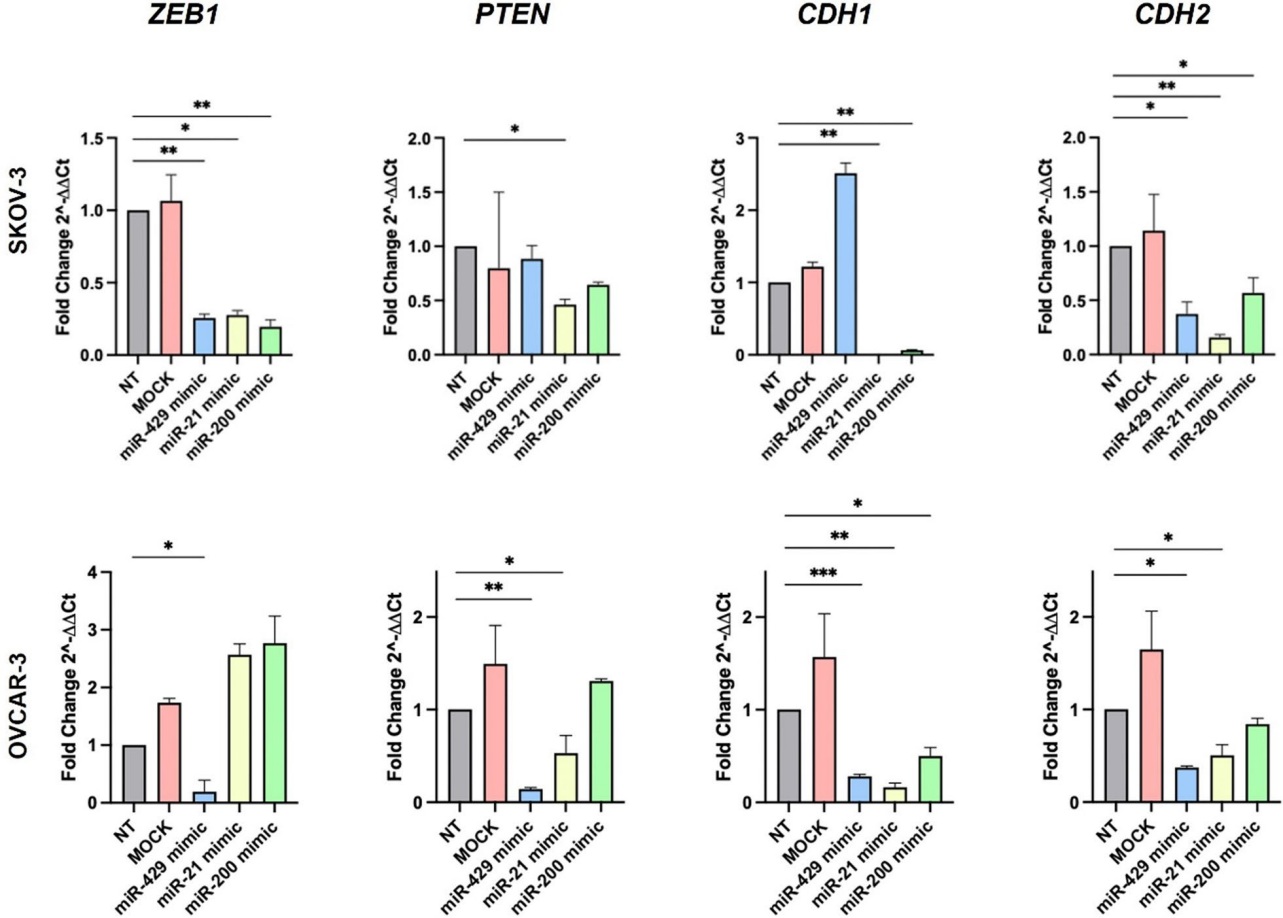
Expression level of *ZEB1, PTEN, CDH1* (E-Cadherin) and* CDH2* (N-Cadherin) mRNA after miRNAs mimics (miR-429 mimic, miR-21 mimic and miR-200 mimic) transfection in SKOV-3, and OVCAR-3; *** = *P* < 0.001; ** = *P* < 0.01: * = *P* < 0.05

Given the potential modulation of EMT highlighted by the functional enrichment analysis, we focused on genes involved in this process (*ZEB1*, *PTEN*, *CDH1* and *CDH2*). High efficiency of mimic transfection was confirmed through RT-PCR in both the cell lines, by evaluating the miRNAs levels (Supplementary Figure S3). The effect of miRNA mimics on the predicted target genes was then evaluated (Fig. 7).

Exogenous over-expression of miR-429 led to a statistically significant decrease in *ZEB1* and *CDH2* mRNA levels in both SKOV-3 and OVCAR-3. Similarly, transfection of miR-200 mimic was correlated with lower *CDH1* mRNA, while miR-21 mimic elicited significant down-regulation of *PTEN*, *CDH1*, and *CDH2*.

### EVs miRNA cargo correlates with OC features and patient prognosis

To assess the clinical relevance of EVs miRNA cargo in ascitic fluid and serum matrices, we analyzed their correlation with CA125 and CA19.9 levels at diagnosis, as well as with *BRCA* status and Peritoneal Cancer Index (PCI) at the time of initial laparoscopy, which quantifies disease burden in the abdomen. The results revealed significant associations between specific miRNAs and CA125 and CA19.9 (Table 3). In particular, miR-200a-3p, miR-200b-3p, miR-200c-3p and miR-429 levels in EVs derived from both ascites and blood were significantly correlated with CA125; similarly, miR-429 levels in EVs derived from both ascites and blood were significantly correlated with CA19.9. No significant results were obtained for *BRCA* status and for PCI (Supplementary Table S6, S7).

**Table 3:** Correlation of miRNA cargo levels from patients’ serum and ascitic fluid with CA125 and CA19.9 at the diagnosis

miRNA	Bodily fluid	CA125		CA19.9	
		Coefficient	*P-value*	Coefficient	*P-value*
miR-181b-5p	AF	0.240	0.067	0.042	0.754
	S	0.197	0.136	0.165	0.216
miR-200a-3p	AF	0.385	**0.003**	0.191	0.151
	S	0.450	** < 0.001**	0.394	**0.002**
miR-200b-3p	AF	0.423	**0.001**	0.197	0.139
	S	0.486	** < 0.001**	0.237	0.073
miR-200c-3p	AF	0.401	**0.002**	0.104	0.436
	S	0.425	**0.001**	0.296	**0.024**
miR-21-5p	AF	0.305	**0.019**	0.191	0.151
	S	0.062	0.643	0.247	0.061
miR-2110	AF	0.126	0.342	0.004	0.979
	S	0.217	0.098	-0.023	0.862
miR-325	AF	0.084	0.524	0.034	0.802
	S	0.322	**0.013**	0.057	0.671
miR-429	AF	0.360	**0.005**	0.270	**0.040**
	S	0.304	**0.020**	0.390	**0.002**
miR-455-5p	AF	0.145	0.272	0.095	0.480
	S	0.364	**0.005**	0.061	0.648

*AF:* ascitic fluid, *S:* serum

**Fig. 8:**
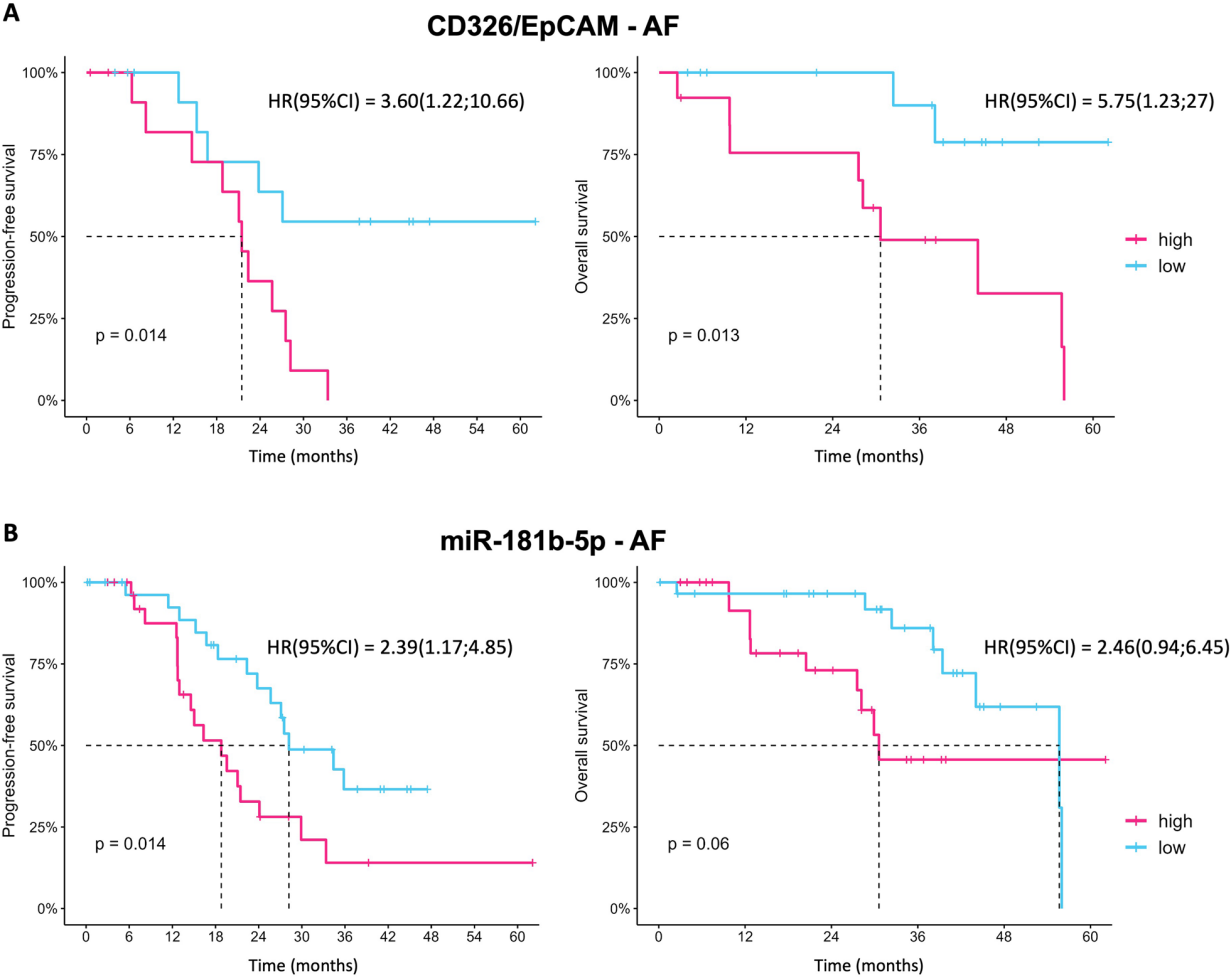
**A** Progression free survival (PFS) and overall survival (OS) of patients with high and low CD326/EpCAM expression on EVs released in ascitic fluid (AF). **B** PFS and OS of patients with high and low miR-181b-5p in ascites-derived EVs

Furthermore, we investigated the prognostic implications of miRNAs and epitopes; we found that higher expression of CD326/EpCAM on ascitic fluid derived EVs was significantly associated with PFS (*P* = 0.014) and OS (*P* = 0.013); in particular, patients with higher expression of EpCAM had a 3.6 fold increased risk to recur earlier and 5.75 fold higher risk to have shorter OS median time, compared with patients with low expression (Fig. 8A). With regard to miRNAs, we observed that higher levels of miR-181b-5p in ascitic fluid derived EVs were significantly associated with PFS (median PFS time: 18.8 vs 28.2 months; *P* = 0.014) and borderline associated with OS (median OS time: 30.6 vs 55.7 months; *P* = 0.060) (Fig. 8B); specifically, patients with high levels of miR-181 had 2.39 fold higher risk to develop recurrence compared with patients with low levels (*P* = 0.016).

## Discussion

This study, representing the first attempt to directly compare EVs derived from blood serum and ascitic fluid in metastatic OC, revealed that ascitic EVs exhibit characteristics indicative of a greater metastatic capacity compared to their blood-derived counterparts. This finding highlights the potential of ascites as a valuable source of tumor-derived EVs, offering promising opportunities for biomarker discovery, which is especially crucial given the high mortality of OC associated with peritoneal metastasis. OC remains one of the most lethal gynecological malignancies, largely due to the lack of early diagnostic biomarkers and the high prevalence of advanced-stage diagnosis (Bachmann 2023). Ascitic fluid accumulation, a common feature of peritoneal metastasis, not only contributes to the pathology but may also play an active role in the dissemination of tumor cells within the peritoneal cavity, self-sustaining the metastatic process (Miceska et al. 2023). In recent years, a growing body of evidence has suggested the crucial role of circulating components, including proteins, CTCs, and EVs in modulating tumor behavior from progression to metastatic development. Recent studies have analyzed the proteome and secretome of OC in biological fluids such as plasma and ascites, identifying potential circulating biomarkers correlated with tumor malignancy and therapeutic response (Qian et al. 2024; Shender et al. 2024). Among circulating components, EVs have attracted particular attention for their role in intercellular communication and metastatic spread, acting as vehicles for the transfer of their molecular cargo (Tian et al. 2022). In this context, the characterization of EVs in readily accessible biological fluids, such as serum and ascites, represents an emerging and promising strategy for cancer diagnosis and drug sensitivity assessment, offering a less invasive alternative to tissue biopsies (Maacha et al. 2019; Ford et al. 2020). Although most initial studies focused on blood-derived EVs, the biofluids closer to the tumor, such as ascites, may be significantly more enriched in tumor-derived substances. Therefore, exploring ascites could be essential for gaining deeper insights into the pathophysiology of metastatic spread and OC recurrence, while also uncovering clinically significant biomarkers. While proteins, such as EpCAM and CD24, were among the first biomarkers studied in EVs derived from malignant ascites (Runz et al. 2007), more recent works have explored the EVs biocargo, focusing particularly on miRNAs. However, the specific differences between EVs detected in ascites and blood have been poorly characterized thus far. Building upon these premises, our study aimed to comparatively analyze EVs released in blood serum and ascites of patients with advanced OC, with the goal of identifying the most promising biofluid for prospective liquid biopsy-based studies in OC.

To achieve this objective, we carried out a comprehensive analysis of EVs in terms of size, surface epitopes expression, and miRNA cargo. Our initial analysis revealed a striking difference in EVs size, with serum-derived EVs significantly smaller compared to ascites-derived EVs, a finding that, to our knowledge, has not been previously reported in a comparative analysis of matched bodily fluids. This size difference is intriguing and may be related to the distinct microenvironments in which EVs are generated. We hypothesize that the greater shear stress experienced by tumor cells and EVs in the bloodstream compared to the more static environment of the ascitic fluid may contribute to the formation of smaller EVs in serum. Indeed, a growing body of literature on the role of biomechanical stress in EVs biogenesis suggests that mechanical forces, such as shear stress, compression, and matrix stiffness, significantly influence their biogenesis, release, and molecular cargo (Thompson and Papoutsakis 2023). These biomechanical cues can induce changes in EV composition, enriching them with oncogenic proteins, lipids, and nucleic acids that promote tumor progression, angiogenesis, and metastasis. To date, no specific studies have been performed to evaluate the role of biomechanical stress in OC, thus no definitive conclusions can be drawn regarding the diverse EVs size in blood serum and ascites. This observed size difference prompted us to further investigate whether EVs from these two sources also differed in their functional characteristics, leading us to analyze their surface epitopes and miRNA cargo.

The analysis of EVs epitopes revealed marked differences in expression levels between ascites and serum EVs, not previously reported in comparative studies. Specifically, ascites-derived EVs exhibited a significantly higher expression of epitopes known to play a crucial role in EMT and metastatic spread. Among these, CD326/EpCAM and CD44 particularly attracted our attention. EpCAM, a transmembrane glycoprotein widely recognized as a “universal” tumor marker for epithelial-derived cancers, is indicative of EVs originating from malignant cells. Several reports, in agreement with our results, showed that elevated levels of EpCAM-positive EVs typically reflect an increased tumor burden and have been correlated with poorer prognosis in various cancer types, including OC (Runz et al. 2007; Jamali et al. 2025). EpCAM is involved in multiple cellular processes, including cell signaling, migration, proliferation, and differentiation. A link between EpCAM expression and the EMT process has recently been highlighted, and it is plausible that the over-expression of EpCAM observed in ascites-derived EVs is related to the invasive capacity of OC in the peritoneal cavity. This is further supported by the fact that, in OC, metastatic spread predominantly occurs via the transcoelomic route, i.e., through the abdominal cavity, rather than hematogenously (Tavsan and Ayar Kayalı 2020; Eslami-S et al. 2020; Patriarca et al. 2012). Similarly, CD44, another over-expressed epitope on ascitic EVs, has been widely implicated in the modulation of EMT, tumor progression, and invasion in several cancer types, including OC (Tavsan and Ayar Kayalı 2020; Eslami-S et al. 2020; Zhou et al. 2012; Tirino et al. 2013). Interestingly, CD44 is also a surface marker of cancer stem cells (CSCs), the subpopulation of tumor cells endowed with stem-like properties that promote tumor initiation, invasive growth, and metastasis formation (Tirino et al. 2013). This suggests that ascites EVs, enriched in these EMT-associated epitopes, may play a direct role in facilitating peritoneal metastasis.

With regard to the analysis of miRNA cargo, miRNA profiling revealed a distinct clustering of samples based on biofluid origin, highlighting unique miRNA signatures in serum and ascitic EVs, as demonstrated by PCA and heatmap analysis. Quantitative analysis identified 98 miRNAs with significantly different abundance between the two EVs populations. The analysis of miRNAs in serum EVs compared to tumor tissue revealed 143 differential expressed miRNAs, while on the other hand, ascitic EVs showed a slightly lower number of deregulated miRNAs compared to tumor (113). This reinforces the idea that ascitic fluid could be more similar to the original tumor and that could represent a valuable source of tumor-related biomarkers. Among the most significant miRNAs, the miR-200 family attracted our attention. This family, including miR-200a, miR-200b, miR-200c, and miR-429, plays a key role in the EMT and in its reverse process mesenchymal-to-epithelial transition (MET) in many epithelial tumors, including OC, by targeting *ZEB1/ZEB2* (Ma et al. 2021; Teng et al. 2016; Cavallari et al. 2021; Wang et al. 2019). Notably, we observed significantly higher levels of miR-200 family members in ascites-derived EVs compared to serum-derived EVs. This finding supports the hypothesis that ascites-derived EVs promote MET in disseminated tumor cells. The miR-200 family directly suppresses the transcription factors ZEB1/ZEB2, which are key repressors of epithelial genes and inducers of EMT (Brabletz and Brabletz 2010; Korpal et al. 2008). While EMT is necessary for the initial dissemination of cancer cells, MET is crucial for the subsequent establishment of metastatic colonies at distant sites. Given the predominantly peritoneal route of metastasis in OC and considering that the patients analyzed in this study presented metastasis, the enrichment of miR-200 family members in ascitic EVs suggests a role in facilitating this secondary MET process.

Indeed, epithelial cells are highly proliferative but non-motile, in contrast to invasive mesenchymal cells that proliferate slowly (Shah et al. 2023). Accordingly, these EVs, potentially through the transfer of specific miRNAs or proteins, may promote peritoneal colonization by inducing an epithelial-like phenotype in disseminated tumor cells.

In addition, our analysis revealed that several circulating miRNAs were significantly associated with CA125 levels, a well-established biomarker for OC diagnosis and disease monitoring. Notably, miR-200 family members exhibited a strong correlation with CA125 expression, reinforcing their potential role as complementary biomarkers for tracking tumor burden and treatment response. This observation is consistent with previous studies suggesting that circulating miRNAs, particularly those encapsulated in EVs, may serve as minimally invasive biomarkers (Yokoi et al. 2018; Zhang et al. 2019).

To further understand the functional implications of the differentially abundant miRNAs, we performed Gene Ontology (GO) enrichment analysis of their predicted target genes. This analysis revealed that the most significantly enriched GO terms were related to EMT and in response to Transforming Growth Factor beta (TGF-β). The enrichment of EMT/MET-related terms is consistent with our findings on epitope expression and miRNA cargo, further supporting the hypothesis that ascites-derived EVs are enriched in factors promoting metastasis. EMT is a critical process in cancer progression, enabling tumor cells to acquire invasive and metastatic capabilities. The enrichment of TGF-β signaling pathways is also highly relevant in the context of OC metastasis, considering that TGF-β is a well-known inducer of EMT and a promoter of invasion in OC (Yeung et al. 2013). Our finding that genes involved in this pathway are enriched targets of differentially abundant miRNAs in EVs, coupled with our in vitro validation, strongly supports the pro-metastatic role of ascitic fluid EVs in OC. Furthermore, the enrichment of extracellular matrix (ECM)-related biological functions among the differentially expressed genes suggests that TGF-β signaling, potentially mediated by ascitic EVs, may also regulate OC development and progression through modulation of the ECM.

Finally, we considered the clinical implications of our findings, showing that miR-181b was significantly associated with patients’ survival. Consistent with previous reports highlighting miR-181b as a cancer-related miRNA (Suszynska et al. 2024; Flores et al. 2017), our results demonstrate a significant correlation between higher miR-181b expression and poorer prognosis in OC patients. This observation suggests that elevated miR-181b levels contribute to a more aggressive tumor phenotype, potentially influencing key pathways involved in disease progression. These findings underscore the prognostic significance of miR-181b in OC and suggest its potential as a valuable biomarker for risk stratification and personalized management of the disease.

Future research should focus on further validating these findings in larger clinical cohorts, elucidating the functional mechanisms beyond the observed differences, and exploring the clinical utility of ascitic EV-derived biomarkers for improved diagnosis, prognosis, and therapeutic monitoring in OC. Finally, while our primary focus was characterizing the differences between ascites- and blood-derived EVs, we acknowledge that a potential limitation of this study is the lack of protein-level validation for the observed mRNA changes. Future studies will prioritize confirming these findings at the protein level using western blot analysis or other appropriate techniques.

## Conclusion

Our study provides compelling evidence that EVs derived from ascitic fluid in metastatic OC patients exhibit distinct characteristics compared to those from blood serum, notably showing features indicative of enhanced metastatic potential. These differences, observed in EVs size, surface epitopes, and miRNA cargo, highlight the importance of considering the tumor microenvironment and the specific biofluid source when studying EVs in cancer. Our findings underscore the potential of ascitic fluid as a rich source of tumor-derived EVs and biomarkers for OC particularly for understanding peritoneal metastasis.

## Supplementary Information

Supplementary material 1.

## Data Availability

MiRNA expression data are deposited into the Gene Expression Omnibus database under accession number GSE278022, https://www.ncbi.nlm.nih.gov/geo/query/acc.cgi?acc=GSE278022.

## References

[CR1] Bachmann C. New achievements from molecular biology and treatment options for refractory/relapsed ovarian cancer-a systematic review. Cancers. 2023;15:5356. 38001616 10.3390/cancers15225356 PMC10669965

[CR2] Bandini E, Rossi T, Scarpi E, et al. Early detection and investigation of extracellular vesicles biomarkers in breast cancer. Front Mol Biosci. 2021;8:732900. 34820420 10.3389/fmolb.2021.732900 PMC8606536

[CR3] Becker A, Thakur BK, Weiss JM, Kim HS, Peinado H, Lyden D. Extracellular vesicles in cancer: cell-to-cell mediators of metastasis. Cancer Cell. 2016;30:836–48. 27960084 10.1016/j.ccell.2016.10.009 PMC5157696

[CR4] Brabletz S, Brabletz T. The ZEB/miR-200 feedback loop-a motor of cellular plasticity in development and cancer? EMBO Rep. 2010;11:670–7. 20706219 10.1038/embor.2010.117 PMC2933868

[CR5] Cavallari I, Ciccarese F, Sharova E, et al. The miR-200 family of microRNAs: fine tuners of epithelial-mesenchymal transition and circulating cancer biomarkers. Cancers. 2021;13:5874. 34884985 10.3390/cancers13235874 PMC8656820

[CR6] Dai W, Zhou J, Chen T. Unraveling the extracellular vesicle network: insights into ovarian cancer metastasis and chemoresistance. Mol Cancer. 2024;23:1–19. 39285475 10.1186/s12943-024-02103-x PMC11404010

[CR7] Ekström K, Crescitelli R, Pétursson HI, Johansson J, Lässer C, Bagge RO. Characterization of surface markers on extracellular vesicles isolated from lymphatic exudate from patients with breast cancer. BMC Cancer. 2022;22:1–17. 35012489 10.1186/s12885-021-08870-w PMC8744234

[CR8] Eslami-S Z, Cortés-Hernández LE, Alix-Panabières C. Epithelial cell adhesion molecule: an anchor to isolate clinically relevant circulating tumor cells. Cells. 2020;9:1–17. 10.3390/cells9081836 PMC7464831 32764280

[CR9] Flores CP, Garcia-Vázquez R, Rincón DG, et al. MicroRNAs driving invasion and metastasis in ovarian cancer: Opportunities for translational medicine (Review). Int J Oncol. 2017;50:1461–76. 28393213 10.3892/ijo.2017.3948

[CR10] Ford CE, Werner B, Hacker NF, Warton K. The untapped potential of ascites in ovarian cancer research and treatment. Br J Cancer. 2020;123:9–16. 32382112 10.1038/s41416-020-0875-x PMC7341795

[CR11] Jabalee J, Towle R, Garnis C. The role of extracellular vesicles in cancer: cargo, function, and therapeutic implications. Cells. 2018;7:93. 30071693 10.3390/cells7080093 PMC6115997

[CR12] Jamali Z, Razipour M, Zargar M, Ghasemnejad-Berenji H, Akrami SM. Ovarian cancer extracellular vesicle biomarkers. Clin Chim Acta. 2025;565:120011. 39437983 10.1016/j.cca.2024.120011

[CR13] Korpal M, Lee ES, Hu G, Kang Y. The miR-200 family inhibits epithelial-mesenchymal transition and cancer cell migration by direct targeting of E-cadherin transcriptional repressors ZEB1 and ZEB2. J Biol Chem. 2008;283:14910–4. 18411277 10.1074/jbc.C800074200 PMC3258899

[CR14] Ma X, Ying Y, Sun J, et al. circKDM4C enhances bladder cancer invasion and metastasis through miR-200bc-3p/ZEB1 axis. Cell Death Discov. 2021;7:1–10. 10.1038/s41420-021-00712-9 PMC8608878 34811353

[CR15] Maacha S, Bhat AA, Jimenez L, Raza A, Haris M, Uddin S, Grivel JC. Extracellular vesicles-mediated intercellular communication: roles in the tumor microenvironment and anti-cancer drug resistance. Mol Cancer. 2019;18:1–16. 30925923 10.1186/s12943-019-0965-7 PMC6441157

[CR16] Micek HM, Rosenstock L, Ma Y, et al. Model of collective detachment in high-grade serous ovarian cancer demonstrates that tumor spheroids produce ECM to support metastatic processes. APL Bioeng. 2023;7:016111. 36875739 10.1063/5.0132254 PMC9977464

[CR17] Miceska S, Skof E, Bucek S, Kuhar CG, Gasljevic G, Smrkolj S, Prevodnik VK. The prognostic significance of tumor-immune microenvironment in ascites of patients with high-grade serous carcinoma. Radiol Oncol. 2023;57:493–506. 38038414 10.2478/raon-2023-0046 PMC10690755

[CR18] Morand S, Devanaboyina M, Staats H, Stanbery L, Nemunaitis J. Ovarian cancer immunotherapy and personalized medicine. Int J Mol Sci. 2021;22:6532. 34207103 10.3390/ijms22126532 PMC8234871

[CR19] Patriarca C, Macchi RM, Marschner AK, Mellstedt H. Epithelial cell adhesion molecule expression (CD326) in cancer: a short review. Cancer Treat Rev. 2012;38:68–75. 21576002 10.1016/j.ctrv.2011.04.002

[CR20] Qian L, Zhu J, Xue Z, et al. Proteomic landscape of epithelial ovarian cancer. Nat Commun. 2024;15:6462. 39085232 10.1038/s41467-024-50786-z PMC11291745

[CR21] Rinnerthaler G, Hackl H, Gampenrieder SP, et al. miR-16-5p is a stably-expressed housekeeping MicroRNA in breast cancer tissues from primary tumors and from metastatic sites. Int J Mol Sci. 2016;17:156. 26821018 10.3390/ijms17020156 PMC4783890

[CR22] Runz S, Keller S, Rupp C, et al. Malignant ascites-derived exosomes of ovarian carcinoma patients contain CD24 and EpCAM. Gynecol Oncol. 2007;107:563–71. 17900673 10.1016/j.ygyno.2007.08.064

[CR23] Shah S, Philipp LM, Giaimo S, Sebens S, Traulsen A, Raatz M. Understanding and leveraging phenotypic plasticity during metastasis formation. Npj Syst Biol Appl. 2023;9:1–11. 37803056 10.1038/s41540-023-00309-1 PMC10558468

[CR24] Shender VO, Anufrieva KS, Shnaider PV, et al. Therapy-induced secretion of spliceosomal components mediates pro-survival crosstalk between ovarian cancer cells. Nat Commun. 2024;15:5237. 38898005 10.1038/s41467-024-49512-6 PMC11187153

[CR25] Suszynska M, Machowska M, Fraszczyk E, Michalczyk M, Philips A, Galka-Marciniak P, Kozlowski P. CMC: Cancer miRNA Census—a list of cancer-related miRNA genes. Nucleic Acids Res. 2024;52:1628–44. 38261968 10.1093/nar/gkae017 PMC10899758

[CR26] Tavsan Z, Ayar Kayalı H. EpCAM-claudin-tetraspanin-modulated ovarian cancer progression and drug resistance. Cell Adh Migr. 2020;14:57–68. 32091301 10.1080/19336918.2020.1732761 PMC7757826

[CR27] Teng Y, Su X, Zhang X, et al. miRNA-200a/c as potential biomarker in epithelial ovarian cancer (EOC): evidence based on miRNA meta-signature and clinical investigations. Oncotarget. 2016;7:81621–33. 27835595 10.18632/oncotarget.13154 PMC5348417

[CR28] Thompson W, Papoutsakis ET. The role of biomechanical stress in extracellular vesicle formation, composition and activity. Biotechnol Adv. 2023;66:108158. 37105240 10.1016/j.biotechadv.2023.108158

[CR29] Tian W, Lei N, Zhou J, et al. Extracellular vesicles in ovarian cancer chemoresistance, metastasis, and immune evasion. Cell Death Dis. 2022;13:1–12. 10.1038/s41419-022-04510-8 PMC8766448 35042862

[CR30] Tirino V, Desiderio V, Paino F, et al. Cancer stem cells in solid tumors: an overview and new approaches for their isolation and characterization. FASEB J. 2013;27:13–24. 23024375 10.1096/fj.12-218222

[CR31] Wang W, Wu LR, Li C, et al. Five serum microRNAs for detection and predicting of ovarian cancer. Eur J Obstet Gynecol Reprod Biol X. 2019;3:100017. 31404211 10.1016/j.eurox.2019.100017 PMC6687444

[CR32] Wang W, Jo HA, Park S, et al. Integrated analysis of ascites and plasma extracellular vesicles identifies a miRNA-based diagnostic signature in ovarian cancer. Cancer Lett. 2022;542:215735. 35569696 10.1016/j.canlet.2022.215735

[CR33] Wiklander OPB, Bostancioglu RB, Welsh JA, et al. Systematic methodological evaluation of a multiplex bead-based flow cytometry assay for detection of extracellular vesicle surface signatures. Front Immunol. 2018;9:378189. 10.3389/fimmu.2018.01326 PMC6008374 29951064

[CR34] Xu R, Greening DW, Zhu HJ, Takahashi N, Simpson RJ. Extracellular vesicle isolation and characterization: toward clinical application. J Clin Invest. 2016;126:1152–62. 27035807 10.1172/JCI81129 PMC4811150

[CR35] Yeung TL, Leung CS, Wong KK, et al. TGF-β Modulates ovarian cancer invasion by upregulating CAF-Derived versican in the tumor microenvironment. Cancer Res. 2013;73:5016–28. 23824740 10.1158/0008-5472.CAN-13-0023 PMC3745588

[CR36] Yokoi A, Matsuzaki J, Yamamoto Y, et al. Integrated extracellular microRNA profiling for ovarian cancer screening. Nat Commun. 2018;9:1–10. 30333487 10.1038/s41467-018-06434-4 PMC6192980

[CR37] Zhang H, Xu S, Liu X. MicroRNA profiling of plasma exosomes from patients with ovarian cancer using high-throughput sequencing. Oncol Lett. 2019;17:5601. 31186782 10.3892/ol.2019.10220 PMC6507395

[CR38] Zhang Q, Ding J, Wang Y, He L, Xue F. Tumor microenvironment manipulates chemoresistance in ovarian cancer (Review). Oncol Rep. 2022;47:102. 35362546 10.3892/or.2022.8313

[CR39] Zhou DX, Liu YX, Xue YH. Expression of CD44v6 and Its association with prognosis in epithelial ovarian carcinomas. Patholog Res Int. 2012;2012:908206. 22482084 10.1155/2012/908206 PMC3317067

