# Supplementary material for: Distinctive features of blood- and ascitic fluid-derived extracellular vesicles in ovarian cancer patients
Source: Mol Med. 2025 Apr 21;31:143. doi: 10.1186/s10020-025-01177-7 (PMC12010555; doi:10.1186/s10020-025-01177-7)

| **Supplementary Table S1: Main clinical and pathological features of OC patients involved in the study** | |
| --- | --- |
|  | **Total N = 60 (%)** |
| **Age**  ≤ 50  > 50 | 13 (21.7%)  47 (78.3%) |
| **Grade**  G1  G2  G3 | 0  0  60 (100%) |
| **FIGO Stage**  I  II  III  IV | 0  2 (3.3%)  45 (75%)  13 (21.7%) |
| **Type of surgery**  PDS  IDS | 28(46.6)  32(53.7) |
| ***BRCA* Status**  WT  BRCA 1/2 MUT  *N/A* | 32 (60.4%)  21 (39.6%)  *7* |

| **Supplementary Table S2: miRNAs significantly deregulated between serum and tumor tissue** | | | | |
| --- | --- | --- | --- | --- |
| **Target** | ***P-value*** | ***Adjusted P-value*** | **Mean ΔCt Serum** | **Mean** **ΔCt Tumor** |
| miR-708-5p | 5.86E-14 | 7.70E-12 | 14.70 | 5.89 |
| miR-214-3p | 5.97E-11 | 1.96E-09 | 8.68 | 1.70 |
| miR-31-5p | 2.17E-10 | 5.70E-09 | 9.20 | 2.66 |
| miR-455-5p | 4.19E-10 | 9.45E-09 | 13.43 | 6.73 |
| miR-224-5p | 4.52E-10 | 9.45E-09 | 7.28 | 2.75 |
| miR-125b-5p | 1.82E-09 | 2.51E-08 | 3.35 | -2.63 |
| miR-455-3p | 1.31E-08 | 1.64E-07 | 11.46 | 4.42 |
| miR-193b-3p | 2.78E-08 | 3.33E-07 | 9.00 | 2.25 |
| miR-181b-5p | 3.38E-08 | 3.86E-07 | 5.86 | 1.90 |
| miR-34a-5p | 1.71E-07 | 1.78E-06 | 9.47 | 1.62 |
| miR-325 | 1.76E-07 | 1.78E-06 | 8.02 | 14.81 |
| miR-452-5p | 1.94E-07 | 1.89E-06 | 11.08 | 6.16 |
| miR-155-5p | 2.93E-07 | 2.58E-06 | 7.37 | 3.50 |
| miR-200b-3p | 3.04E-07 | 2.58E-06 | 7.06 | -0.25 |
| miR-193a-5p | 5.10E-07 | 4.06E-06 | 8.89 | 4.53 |
| miR-223-3p | 1.05E-06 | 8.09E-06 | -1.52 | 2.18 |
| miR-502-5p | 1.16E-06 | 8.73E-06 | 14.31 | 8.07 |
| miR-195-5p | 1.28E-06 | 9.37E-06 | 7.20 | 3.59 |
| miR-148a-3p | 1.51E-06 | 1.05E-05 | 5.18 | 2.49 |
| miR-548a-3p | 2.61E-06 | 1.63E-05 | 10.62 | 14.92 |
| let-7b-5p | 2.99E-06 | 1.83E-05 | 9.12 | 5.77 |
| miR-501-5p | 5.11E-06 | 2.99E-05 | 11.65 | 6.84 |
| miR-450a-5p | 5.31E-06 | 3.04E-05 | 11.90 | 6.19 |
| miR-200c-3p | 5.82E-06 | 3.26E-05 | 9.87 | 1.54 |
| miR-125a-5p | 7.66E-06 | 4.20E-05 | 3.70 | -0.61 |
| miR-210-3p | 9.24E-06 | 4.86E-05 | 5.79 | 2.52 |
| miR-34c-5p | 1.80E-05 | 9.11E-05 | 12.31 | 7.26 |
| miR-424-5p | 2.45E-05 | 1.19E-04 | 8.09 | 4.78 |
| miR-500a-5p | 2.74E-05 | 1.31E-04 | 8.71 | 5.29 |
| miR-887-3p | 3.07E-05 | 1.44E-04 | 12.72 | 6.64 |
| miR-99b-5p | 3.76E-05 | 1.73E-04 | 7.02 | 3.23 |
| miR-885-5p | 4.30E-05 | 1.92E-04 | 11.95 | 7.69 |
| miR-21-5p | 6.22E-05 | 2.68E-04 | 1.33 | -0.57 |
| miR-362-5p | 7.10E-05 | 2.96E-04 | 7.80 | 4.45 |
| miR-222-3p | 8.75E-05 | 3.60E-04 | 5.27 | 2.75 |
| miR-874-3p | 1.02E-04 | 4.13E-04 | 10.37 | 6.51 |
| miR-505-3p | 1.10E-04 | 4.38E-04 | 7.76 | 4.85 |
| miR-361-5p | 1.21E-04 | 4.77E-04 | 4.54 | 2.08 |
| miR-92b-3p | 1.79E-04 | 6.74E-04 | 2.62 | 0.72 |
| miR-24-3p | 2.03E-04 | 7.42E-04 | 1.94 | -0.30 |
| miR-493-3p | 2.09E-04 | 7.54E-04 | 14.76 | 11.51 |
| miR-204-5p | 2.46E-04 | 8.62E-04 | 10.59 | 5.16 |
| miR-342-3p | 4.08E-04 | 1.36E-03 | 4.55 | 1.84 |
| miR-153-3p | 5.11E-04 | 1.66E-03 | 8.37 | 12.53 |
| miR-142-3p | 6.04E-04 | 1.94E-03 | 2.46 | 7.14 |
| miR-152-3p | 6.35E-04 | 1.99E-03 | 8.61 | 4.97 |
| miR-181a-5p | 7.13E-04 | 2.15E-03 | 2.77 | 0.69 |
| miR-130a-3p | 8.06E-04 | 2.41E-03 | 3.57 | 1.14 |
| miR-130b-3p | 8.56E-04 | 2.53E-03 | 7.29 | 3.93 |
| miR-503-5p | 1.17E-03 | 3.41E-03 | 9.79 | 7.46 |
| miR-380-3p | 1.19E-03 | 3.45E-03 | 6.24 | 9.50 |
| miR-551b-3p | 1.27E-03 | 3.63E-03 | 11.56 | 7.61 |
| miR-92a-3p | 1.31E-03 | 3.71E-03 | 1.23 | -0.77 |
| miR-429 | 1.57E-03 | 4.39E-03 | 8.33 | 4.87 |
| miR-185-5p | 1.84E-03 | 5.05E-03 | 4.21 | 6.28 |
| miR-448 | 2.56E-03 | 6.81E-03 | 8.19 | 10.69 |
| miR-451a | 3.00E-03 | 7.88E-03 | -3.19 | 1.12 |
| miR-143-3p | 3.13E-03 | 8.15E-03 | 6.33 | 2.02 |
| miR-181c-5p | 3.16E-03 | 8.16E-03 | 4.38 | 2.39 |
| miR-653-5p | 3.22E-03 | 8.22E-03 | 8.98 | 12.37 |
| miR-26a-5p | 3.55E-03 | 8.99E-03 | 0.61 | -1.53 |
| miR-10a-5p | 4.50E-03 | 1.12E-02 | 5.28 | 2.05 |
| miR-486-5p | 5.24E-03 | 1.28E-02 | 1.31 | 4.49 |
| miR-148b-3p | 5.32E-03 | 1.28E-02 | 4.78 | 3.14 |
| miR-128-3p | 5.40E-03 | 1.29E-02 | 5.36 | 3.31 |
| miR-145-5p | 6.54E-03 | 1.54E-02 | 2.70 | -0.33 |
| miR-25-3p | 8.36E-03 | 1.90E-02 | 1.40 | -0.39 |
| miR-29b-3p | 8.43E-03 | 1.90E-02 | 4.23 | 2.91 |
| miR-135a-5p | 9.17E-03 | 2.04E-02 | 11.14 | 7.45 |
| miR-660-5p | 9.78E-03 | 2.16E-02 | 6.90 | 4.96 |
| miR-181d-5p | 1.20E-02 | 2.52E-02 | 10.08 | 7.63 |
| miR-127-3p | 1.32E-02 | 2.75E-02 | 6.33 | 3.43 |
| miR-199a-3p/miR-199b-3p | 1.41E-02 | 2.91E-02 | 2.02 | 0.20 |
| miR-338-3p | 1.49E-02 | 3.07E-02 | 6.83 | 8.93 |
| miR-103a-2-5p | 1.55E-02 | 3.17E-02 | 9.84 | 7.26 |
| miR-518e-3p | 1.61E-02 | 3.25E-02 | 13.07 | 15.11 |
| let-7e-5p | 1.65E-02 | 3.32E-02 | 5.16 | 1.97 |
| miR-29a-3p | 1.71E-02 | 3.38E-02 | 3.64 | 0.10 |
| miR-28-3p | 1.74E-02 | 3.42E-02 | 6.55 | 3.93 |
| miR-146a-5p | 1.79E-02 | 3.45E-02 | 1.92 | 3.88 |
| miR-19a-3p | 1.79E-02 | 3.45E-02 | 4.35 | 8.14 |
| miR-345-5p | 1.98E-02 | 3.75E-02 | 8.10 | 4.95 |
| miR-598-3p | 2.06E-02 | 3.87E-02 | 9.98 | 8.52 |
| miR-485-5p | 2.27E-02 | 4.24E-02 | 13.05 | 10.34 |
| miR-126-3p | 2.49E-02 | 4.61E-02 | -0.63 | 1.73 |
| miR-302b-3p | 2.61E-02 | 4.80E-02 | 14.11 | 16.46 |

| **Supplementary Table S3: miRNAs significantly deregulated between ascitic fluid and tumor tissue** | | | | |
| --- | --- | --- | --- | --- |
| **Target** | ***P-value*** | ***Adjusted P-value*** | **Mean ΔCt Ascitic fluid** | **Mean ΔCt Tumor** |
| miR-325 | 1.23E-13 | 1.62E-11 | 3.29 | 14.81 |
| miR-448 | 4.05E-09 | 2.13E-07 | 5.01 | 10.69 |
| miR-653-5p | 6.13E-09 | 2.69E-07 | 4.58 | 12.37 |
| miR-199a-5p | 2.56E-08 | 7.49E-07 | 11.37 | 2.43 |
| miR-302b-3p | 2.42E-07 | 4.90E-06 | 10.26 | 16.46 |
| miR-153-3p | 3.34E-06 | 3.66E-05 | 6.63 | 12.53 |
| miR-380-3p | 3.60E-06 | 3.79E-05 | 4.52 | 9.50 |
| miR-199a-3p/miR-199b-3p | 6.11E-06 | 6.18E-05 | 3.86 | 0.20 |
| miR-486-5p | 9.56E-06 | 8.67E-05 | 9.91 | 4.49 |
| miR-214-3p | 1.18E-05 | 1.03E-04 | 5.73 | 1.70 |
| miR-708-5p | 1.37E-05 | 1.16E-04 | 9.90 | 5.89 |
| miR-570-3p | 1.47E-05 | 1.17E-04 | 10.50 | 14.48 |
| hiR-328-3p | 3.08E-05 | 2.19E-04 | 9.19 | 5.47 |
| let-7e-5p | 4.84E-05 | 3.18E-04 | 7.73 | 1.97 |
| miR-101-3p | 7.66E-05 | 4.79E-04 | 2.91 | 5.85 |
| miR-126-3p | 1.42E-04 | 8.12E-04 | 5.97 | 1.73 |
| miR-582-5p | 2.32E-04 | 1.25E-03 | 12.35 | 6.74 |
| miR-28-3p | 3.79E-04 | 1.95E-03 | 8.01 | 3.93 |
| miR-181a-5p | 5.37E-04 | 2.52E-03 | 2.82 | 0.69 |
| miR-433-3p | 6.83E-04 | 3.15E-03 | 12.26 | 8.01 |
| miR-376a-3p | 7.58E-04 | 3.38E-03 | 6.17 | 8.92 |
| miR-30c-5p | 8.47E-04 | 3.71E-03 | 3.76 | 0.35 |
| miR-29b-3p | 9.17E-04 | 3.95E-03 | 1.20 | 2.91 |
| miR-187-3p | 9.34E-04 | 3.96E-03 | 9.11 | 4.91 |
| miR-629-5p | 1.11E-03 | 4.62E-03 | 6.57 | 9.23 |
| miR-1-3p | 1.25E-03 | 5.06E-03 | 2.56 | 8.12 |
| miR-92b-3p | 1.43E-03 | 5.60E-03 | 2.30 | 0.72 |
| miR-143-3p | 1.61E-03 | 6.03E-03 | 6.65 | 2.02 |
| miR-422a | 1.85E-03 | 6.77E-03 | 11.01 | 8.25 |
| miR-92a-3p | 2.00E-03 | 7.20E-03 | 1.14 | -0.77 |
| miR-363-3p | 2.03E-03 | 7.21E-03 | 7.85 | 5.39 |
| miR-424-5p | 3.15E-03 | 1.09E-02 | 6.97 | 4.78 |
| miR-181b-5p | 3.30E-03 | 1.13E-02 | 3.75 | 1.90 |
| miR-21-5p | 3.90E-03 | 1.30E-02 | -1.89 | -0.57 |
| miR-744-5p | 4.03E-03 | 1.32E-02 | 6.63 | 4.63 |
| miR-503-5p | 4.28E-03 | 1.39E-02 | 9.48 | 7.46 |
| miR-155-5p | 4.38E-03 | 1.39E-02 | 5.43 | 3.50 |
| miR-505-3p | 4.39E-03 | 1.39E-02 | 6.91 | 4.85 |
| miR-342-3p | 4.56E-03 | 1.43E-02 | 3.96 | 1.84 |
| miR-146a-5p | 4.64E-03 | 1.43E-02 | 1.50 | 3.88 |
| miR-34a-5p | 6.63E-03 | 1.93E-02 | 5.24 | 1.63 |
| miR-190a-5p | 6.64E-03 | 1.93E-02 | 6.56 | 10.56 |
| miR-145-5p | 8.04E-03 | 2.27E-02 | 2.62 | -0.33 |
| miR-181c-5p | 8.51E-03 | 2.36E-02 | 4.15 | 2.39 |
| miR-532-3p | 8.84E-03 | 2.41E-02 | 9.71 | 6.11 |
| miR-324-5p | 9.03E-03 | 2.42E-02 | 5.35 | 3.25 |
| miR-103a-3p | 9.99E-03 | 2.63E-02 | 5.91 | 3.08 |
| miR-499a-5p | 1.04E-02 | 2.70E-02 | 10.27 | 13.01 |
| miR-152-3p | 1.46E-02 | 3.63E-02 | 7.49 | 4.97 |
| miR-502-3p | 1.46E-02 | 3.63E-02 | 1.24 | 4.09 |
| miR-133a-3p | 1.58E-02 | 3.88E-02 | 11.11 | 7.28 |
| miR-421 | 1.63E-02 | 3.95E-02 | 9.85 | 6.97 |
| miR-128-3p | 1.64E-02 | 3.95E-02 | 5.06 | 3.31 |
| miR-25-3p | 1.68E-02 | 3.98E-02 | 1.22 | -0.39 |
| miR-221-3p | 1.86E-02 | 4.37E-02 | -1.69 | -0.15 |

| **Supplementary Table S4: miRNAs significantly deregulated between normal and tumor tissue** | | | | |
| --- | --- | --- | --- | --- |
| **Target** | **P-value** | **Adjusted P-value** | **Mean ΔCt Tumor** | **Mean ΔCt Normal Tissue** |
| miR-383-5p | 7.16E-11 | 1.86E-08 | 16.36 | 9.14 |
| miR-149-5p | 1.16E-06 | 1.50E-04 | 5.60 | -0.02 |
| miR-21-5p | 1.41E-05 | 1.22E-03 | -0.57 | 1.90 |
| miR-381-3p | 2.52E-05 | 1.64E-03 | 8.38 | 12.54 |
| miR-302b-3p | 3.47E-05 | 1.81E-03 | 16.46 | 11.59 |
| miR-548am-5p/miR-548c-5p/miR-548o-5p | 5.89E-05 | 2.55E-03 | 16.39 | 12.99 |
| miR-486-5p | 6.90E-05 | 2.56E-03 | 4.49 | -0.43 |
| miR-145-5p | 8.54E-05 | 2.78E-03 | -0.33 | -4.99 |
| miR-106b-5p | 1.82E-04 | 5.05E-03 | 4.10 | 10.15 |
| miR-615-3p | 2.32E-04 | 5.05E-03 | 8.90 | 5.52 |
| miR-193b-3p | 2.38E-04 | 5.05E-03 | 2.25 | -1.95 |
| miR-135b-5p | 2.51E-04 | 5.05E-03 | 5.49 | 11.35 |
| miR-210-3p | 2.53E-04 | 5.05E-03 | 2.52 | -0.17 |
| miR-200a-3p | 2.76E-04 | 5.12E-03 | 1.60 | 6.55 |
| miR-130a-3p | 3.70E-04 | 5.53E-03 | 1.14 | 3.81 |
| miR-155-5p | 3.89E-04 | 5.53E-03 | 3.50 | 6.12 |
| miR-130b-3p | 4.26E-04 | 5.53E-03 | 3.93 | 7.60 |
| miR-31-5p | 4.29E-04 | 5.53E-03 | 2.66 | 5.94 |
| miR-374b-5p | 4.31E-04 | 5.53E-03 | 6.97 | 9.97 |
| miR-205-5p | 4.36E-04 | 5.53E-03 | 3.47 | -4.54 |
| miR-545-3p | 4.57E-04 | 5.53E-03 | 16.37 | 12.71 |
| miR-338-3p | 4.68E-04 | 5.53E-03 | 8.93 | 5.72 |
| miR-450a-5p | 6.11E-04 | 6.90E-03 | 6.19 | 10.51 |
| miR-301a-3p | 6.61E-04 | 7.16E-03 | 8.91 | 12.21 |
| miR-135a-5p | 7.55E-04 | 7.85E-03 | 7.45 | 12.48 |
| miR-301b-3p | 1.43E-03 | 1.43E-02 | 7.84 | 12.44 |
| miR-518e-3p | 1.51E-03 | 1.43E-02 | 15.11 | 12.09 |
| miR-375 | 1.54E-03 | 1.43E-02 | 7.47 | 1.99 |
| miR-744-5p | 1.62E-03 | 1.45E-02 | 4.63 | 2.31 |
| miR-299-5p | 1.67E-03 | 1.45E-02 | 8.82 | 11.73 |
| miR-328-3p | 1.78E-03 | 1.49E-02 | 5.47 | 2.70 |
| miR-376a-3p | 1.89E-03 | 1.53E-02 | 8.92 | 5.13 |
| miR-203a-3p | 1.96E-03 | 1.54E-02 | 8.15 | 3.05 |
| miR-551b-3p | 2.09E-03 | 1.60E-02 | 7.61 | 11.56 |
| miR-582-5p | 2.52E-03 | 1.86E-02 | 6.74 | 11.42 |
| miR-423-5p | 2.57E-03 | 1.86E-02 | 1.26 | -0.94 |
| miR-214-3p | 2.73E-03 | 1.92E-02 | 1.70 | -0.95 |
| miR-122-5p | 3.77E-03 | 2.58E-02 | 16.59 | 13.18 |
| miR-208b-3p | 4.45E-03 | 2.96E-02 | 15.82 | 12.95 |
| miR-181b-5p | 5.00E-03 | 3.25E-02 | 1.90 | 0.05 |
| miR-185-5p | 5.72E-03 | 3.63E-02 | 6.28 | 4.33 |
| miR-92a-3p | 6.15E-03 | 3.74E-02 | -0.77 | -2.53 |
| miR-320a | 6.18E-03 | 3.74E-02 | 2.51 | 0.22 |
| miR-193a-5p | 6.68E-03 | 3.95E-02 | 4.53 | 2.32 |
| miR-335-5p | 7.88E-03 | 4.55E-02 | 4.15 | 8.75 |
| miR-369-3p | 8.38E-03 | 4.74E-02 | 8.05 | 12.48 |

| **Supplementary Table S5. Predicted target genes of the significantly deregulated miRNAs** | |
| --- | --- |
| **miR-181b-5p** | |
| **Target gene** | **Gene name** |
| *ZNF268* | zinc finger protein 268 |
| *OSBPL3* | oxysterol binding protein-like 3 |
| *GSKIP* | GSK3B interacting protein |
| *ZFP36L1* | ZFP36 ring finger protein-like 1 |
| *C2orf69* | chromosome 2 open reading frame 69 |
| *CBX7* | chromobox homolog 7 |
| *BCL2* | B-cell CLL/lymphoma 2 |
| *PLAG1* | pleiomorphic adenoma gene 1 |
| *SLC25A37* | solute carrier family 25 (mitochondrial iron transporter). member 37 |
| *DDX52* | DEAD (Asp-Glu-Ala-Asp) box polypeptide 52 |
| *ZFP69B* | ZFP69 zinc finger protein B |
| *TMCC1* | transmembrane and coiled-coil domain family 1 |
| *FSD1L* | fibronectin type III and SPRY domain containing 1-like |
| *RPS6KA3* | ribosomal protein S6 kinase. 90kDa. polypeptide 3 |
| *KIAA1551* | KIAA1551 |
| **miR-200a-3p** | |
| **Target gene** | **Gene name** |
| *GATA6* | GATA binding protein 6 |
| *SPAG9* | sperm associated antigen 9 |
| *UBASH3B* | ubiquitin associated and SH3 domain containing B |
| *CDV3* | CDV3 homolog (mouse) |
| *OGT* | O-linked N-acetylglucosamine (GlcNAc) transferase |
| **miR-200b-3p** | |
| **Target gene** | **Gene name** |
| *ERRFI1* | ERBB receptor feedback inhibitor 1 |
| *ZEB2* | zinc finger E-box binding homeobox 2 |
| *ZEB1* | zinc finger E-box binding homeobox 1 |
| *MSN* | moesin |
| *JUN* | jun proto-oncogene |
| *RAB21* | RAB21. member RAS oncogene family |
| *WASF3* | WAS protein family. member 3 |
| *DNAJB9* | DnaJ (Hsp40) homolog. subfamily B. member 9 |
| *DNMT3B* | DNA (cytosine-5-)-methyltransferase 3 beta |
| *MYB* | v-myb avian myeloblastosis viral oncogene homolog |
| *RNF2* | ring finger protein 2 |
| *RHOA* | ras homolog family member A |
| *PIN1* | peptidylprolyl cis/trans isomerase. NIMA-interacting 1 |
| *RND3* | Rho family GTPase 3 |
| *NLGN4X* | neuroligin 4. X-linked |
| *RPS6KB1* | ribosomal protein S6 kinase. 70kDa. polypeptide 1 |
| *FOXG1* | forkhead box G1 |
| *NRBP1* | nuclear receptor binding protein 1 |
| *LOX* | lysyl oxidase |
| *GATA4* | GATA binding protein 4 |
| **miR-200c-3p** | |
| **Target gene** | **Gene name** |
| *KDR* | kinase insert domain receptor (a type III receptor tyrosine kinase) |
| *BAP1* | BRCA1 associated protein-1 (ubiquitin carboxy-terminal hydrolase) |
| **miR-21-5p** | |
| **Target gene** | **Gene name** |
| *ZNF367* | zinc finger protein 367 |
| *KRIT1* | KRIT1. ankyrin repeat containing |
| *IL12A* | interleukin 12A (natural killer cell stimulatory factor 1. cytotoxic lymphocyte maturation factor 1. p35) |
| *FASLG* | Fas ligand (TNF superfamily. member 6) |
| *CCL1* | chemokine (C-C motif) ligand 1 |
| *GPR64* | G protein-coupled receptor 64 |
| *PTEN* | phosphatase and tensin homolog |
| *PLEKHA1* | pleckstrin homology domain containing. family A (phosphoinositide binding specific) member 1 |
| *YOD1* | YOD1 deubiquitinase |
| *PELI1* | pellino E3 ubiquitin protein ligase 1 |
| *TGFBI* | transforming growth factor. beta-induced. |
| *SKP2* | S-phase kinase-associated protein 2. E3 ubiquitin protein ligase |
| *NTF3* | neurotrophin 3 |
| *TIMP3* | TIMP metallopeptidase inhibitor 3 |
| *SMAD7* | SMAD family member 7 |
| *MSH2* | mutS homolog 2 |
| *SATB1* | SATB homeobox 1 |
| *RP2* | retinitis pigmentosa 2 (X-linked recessive) |
| *RTN4* | reticulon 4 |
| *PPP1R3B* | protein phosphatase 1. regulatory subunit 3B |
| **miR-2110** | |
| **Target gene** | **Gene name** |
| *TMEM69* | transmembrane protein 69 |
| *MARCKSL1* | MARCKS-like 1 |
| *TPP1* | tripeptidyl peptidase I |
| *PTPN14* | protein tyrosine phosphatase. non-receptor type 14 |
| *MAX* | MYC associated factor X |
| *VAT1* | vesicle amine transport 1 |
| *DYNAP* | dynactin associated protein |
| *TRIM21* | tripartite motif containing 21 |
| *SERINC1* | serine incorporator 1 |
| *E2F3* | E2F transcription factor 3 |
| *TRAM1* | translocation associated membrane protein 1 |
| *NKIRAS2* | NFKB inhibitor interacting Ras-like 2 |
| *HNRNPUL1* | heterogeneous nuclear ribonucleoprotein U-like 1 |
| *STS* | steroid sulfatase (microsomal). isozyme S |
| *MAPK1* | mitogen-activated protein kinase 1 |
| *CCDC137* | coiled-coil domain containing 137 |
| *MARK2* | MAP/microtubule affinity-regulating kinase 2 |
| *RPRD2* | regulation of nuclear pre-mRNA domain containing 2 |
| *SAT2* | spermidine/spermine N1-acetyltransferase family member 2 |
| *AR* | androgen receptor |
| *SGPL1* | sphingosine-1-phosphate lyase 1 |
| **miR-325** | |
| **Target gene** | **Gene name** |
| *TARBP2* | TAR (HIV-1) RNA binding protein 2 |
| *FAM60A* | family with sequence similarity 60. member A |
| *LAX1* | lymphocyte transmembrane adaptor 1 |
| *ABT1* | activator of basal transcription 1 |
| *CBX4* | chromobox homolog 4 |
| *RBMS1* | RNA binding motif. single stranded interacting protein 1 |
| *TRIM14* | tripartite motif containing 14 |
| *ARSJ* | arylsulfatase family. member J |
| *FBXO21* | F-box protein 21 |
| *NPNT* | nephronectin |
| *PRPF38A* | pre-mRNA processing factor 38A |
| *AGO1* | argonaute RISC catalytic component 1 |
| *CREBBP* | CREB binding protein |
| *TRIM33* | tripartite motif containing 33 |
| *ZNF573* | zinc finger protein 573 |
| *ZNF460* | zinc finger protein 460 |
| *LZIC* | leucine zipper and CTNNBIP1 domain containing |
| *MMP16* | matrix metallopeptidase 16 (membrane-inserted) |
| *PHAX* | phosphorylated adaptor for RNA export |
| *RAB10* | RAB10. member RAS oncogene family |
| **miR-429** | |
| **Target gene** | **Gene name** |
| *ZEB1* | zinc finger E-box binding homeobox 1 |
| *TCEB1* | transcription elongation factor B (SIII). polypeptide 1 (15kDa. elongin C) |
| *PMAIP1* | phorbol-12-myristate-13-acetate-induced protein 1 |
| *ZFPM2* | zinc finger protein. FOG family member 2 |
| *RASSF8* | Ras association (RalGDS/AF-6) domain family (N-terminal) member 8 |
| *ABI2* | abl-interactor 2 |
| *CDKN1B* | cyclin-dependent kinase inhibitor 1B (p27. Kip1) |
| *SOX2* | SRY (sex determining region Y)-box 2 |
| *UBE2D1* | ubiquitin-conjugating enzyme E2D 1 |
| *FRS2* | fibroblast growth factor receptor substrate 2 |
| *TIMP2* | TIMP metallopeptidase inhibitor 2 |
| *UNC119B* | unc-119 homolog B (C. elegans) |
| *DENND5B* | DENN/MADD domain containing 5B |
| *PTEN* | phosphatase and tensin homolog |
| *SEC24A* | SEC24 family. member A (S. cerevisiae) |
| **miR-455-5p** | |
| **Target gene** | **Gene name** |
| *IPO7* | importin 7 |
| *MYLIP* | myosin regulatory light chain interacting protein |
| *SOX11* | SRY (sex determining region Y)-box 11 |
| *USP3* | ubiquitin specific peptidase 3 |
| *ZFAND5* | zinc finger. AN1-type domain 5 |
| *UBASH3B* | ubiquitin associated and SH3 domain containing B |
| *KLHL15* | kelch-like family member 15 |
| *DYRK1A* | dual-specificity tyrosine-(Y)-phosphorylation regulated kinase 1A |
| *CDKN1B* | cyclin-dependent kinase inhibitor 1B (p27. Kip1) |
| *TRIM33* | tripartite motif containing 33 |
| *PATL1* | protein associated with topoisomerase II homolog 1 (yeast) |
| *DDX3X* | DEAD (Asp-Glu-Ala-Asp) box helicase 3. X-linked |
| *ATP13A3* | ATPase type 13A3 |
| *KPNA3* | karyopherin alpha 3 (importin alpha 4) |
| *PLEKHA6* | pleckstrin homology domain containing. family A member 6 |

| **Supplementary Table S6. Analysis of miRNA cargo levels based on patients’ *BRCA* status and peritoneal cancer index (PCI)** | | | | | | |
| --- | --- | --- | --- | --- | --- | --- |
|  |  | **BRCA status**  **Median (IQR)** | | | **PCI** | |
| **miRNA** | **Bodily fluid** | **WT** | **mutated** | ***P-value*** | **coefficient** | ***P-value*** |
| miR-181b-5p | AF | -2.59(-3.67;-1.41) | -3.36(-5.94;-2.04) | 0.225 | 0.004 | 0.980 |
|  | S | -5.22(-6.41;-4.85) | -5.47(-6.09;-4.07) | 0.709 | -0.063 | 0.687 |
| miR-200a-3p | AF | -1.36(-2.53;-0.09) | -1.01(-2.91;-0.4) | 0.796 | 0.003 | 0.987 |
|  | S | -7.68(-9;-6.57) | -8.16(-9.83;-6.45) | 0.631 | -0.084 | 0.591 |
| miR-200b-3p | AF | -0.01(-2.24;1.15) | 0.11(-1.04;1.25) | 0.853 | -0.029 | 0.854 |
|  | S | -8.52(-10.95;-5.08) | -8.66(-10.68;-5.78) | 0.698 | -0.017 | 0.915 |
| miR-200c-3p | AF | -0.64(-2.83;0.51) | -1.66(-4.16;-0.31) | 0.326 | -0.049 | 0.755 |
|  | S | -6.67(-8.28;-4.96) | -7.28(-8.12;-5.72) | 0.657 | -0.034 | 0.828 |
| miR-21-5p | AF | 3.13(2.09;3.51) | 1.93(1.53;3.92) | 0.579 | -0.113 | 0.469 |
|  | S | -1.31(-1.63;-0.9) | -1.32(-1.99;-0.86) | 0.526 | 0.130 | 0.406 |
| miR-2110 | AF | -4.58(-6.57;-3.63) | -5(-6.96;-1.65) | 0.782 | -0.237 | 0.126 |
|  | S | -8.14(-9.91;-7.33) | -8.08(-9.29;-6.28) | 0.426 | -0.239 | 0.124 |
| miR-325 | AF | -3.85(-7.86;-0.87) | -7.07(-10.76;-2.62) | 0.097 | 0.002 | 0.991 |
|  | S | -10.58(-13.53;-7.66) | -11.52(-13.2;-7.2) | 0.787 | -0.156 | 0.319 |
| miR-429 | AF | -2.74(-4.31;-0.73) | -2.09(-3.86;-1.5) | 0.808 | 0 | 0.998 |
|  | S | -10.14(-12.71;-7.22) | -10.55(-12.03;-7.22) | 0.853 | -0.252 | 0.103 |
| miR-455-5p | AF | -5.77(-7.2;-4.52) | -5.13(-7.34;-4.11) | 0.628 | -0.010 | 0.948 |
|  | S | -13.64(-14.77;-11.7) | -12.35(-13.75;-11.27 | 0.176 | -0.010 | 0.950 |

**IQR: interquartile range**

| **Supplementary Table S7. Analysis of miRNA cargo levels based on patients’ peritoneal cancer index (PCI), using a cut-off of 20** | | | | | |
| --- | --- | --- | --- | --- | --- |
|  |  | **PCI**  **Median (IQR)** | | | |
| **miRNA** | **Bodily fluid** | **≤20** | **>20** | ***P-value*** |  |
| miR-181b-5p | AF | -3.24(-4.42;-1.38) | -2.93(-5.7;-1.75) | 0.667 |  |
|  | S | -5.39(-6.23;-4.65) | -4.91(-5.9;-4.39) | 0.604 |  |
| miR-200a-3p | AF | -1.51(-2.96;-0.29) | -0.94(-2.71;-0.38) | 0.734 |  |
|  | S | -7.76(-9.22;-6.64) | -7.58(-9.07;-6.38) | 0.960 |  |
| miR-200b-3p | AF | -0.3(-2.57;1.28) | 0.36(-0.8;0.93) | 0.973 |  |
|  | S | -8.75(-11.89;-6.29) | -7.94(-9.77;-5.32) | 0.148 |  |
| miR-200c-3p | AF | -1.3(-3.45;0.16) | -0.82(-3.84;-0.36) | 0.565 |  |
|  | S | -7.12(-8.47;-5.4) | -6.44(-7.73;-4.78) | 0.373 |  |
| miR-21-5p | AF | 2.59(1.64;3.35) | 2.41(1.63;3.81) | 0.977 |  |
|  | S | -1.45(-2.09;-1.1) | -1.13(-1.71;-0.7) | 0.089 |  |
| miR-2110 | AF | -4.64(-6.42;-3.57) | -5.34(-7.22;-3.39) | 0.727 |  |
|  | S | -8.03(-9.99;-7.42) | -7.44(-8.99;-6.09) | 0.262 |  |
| miR-325 | AF | -3.98(-7.49;-1.4) | -8.1(-10.63;-2.09) | 0.136 |  |
|  | S | -10.94(-14.28;-8.88) | -11.52(-12.39;-7.02) | 0.409 |  |
| miR-429 | AF | -3(-3.98;-1.35) | -1.85(-3.52;-0.89) | 0.594 |  |
|  | S | -11.63(-13.14;-8.41) | -10.12(-10.8;-7.03) | 0.189 |  |
| miR-455-5p | AF | -5.31(-7.02;-4.07) | -6.37(-7.59;-4.62) | 0.069 |  |
|  | S | -13.6(-14.79;-10.66) | -12.06(-13.66;-11.43) | 0.527 |  |

**IQR: interquartile range; PCI: peritoneal cancer index**

**Supplementary Figures**

**Supplementary Figure S1: General workflow of the study**


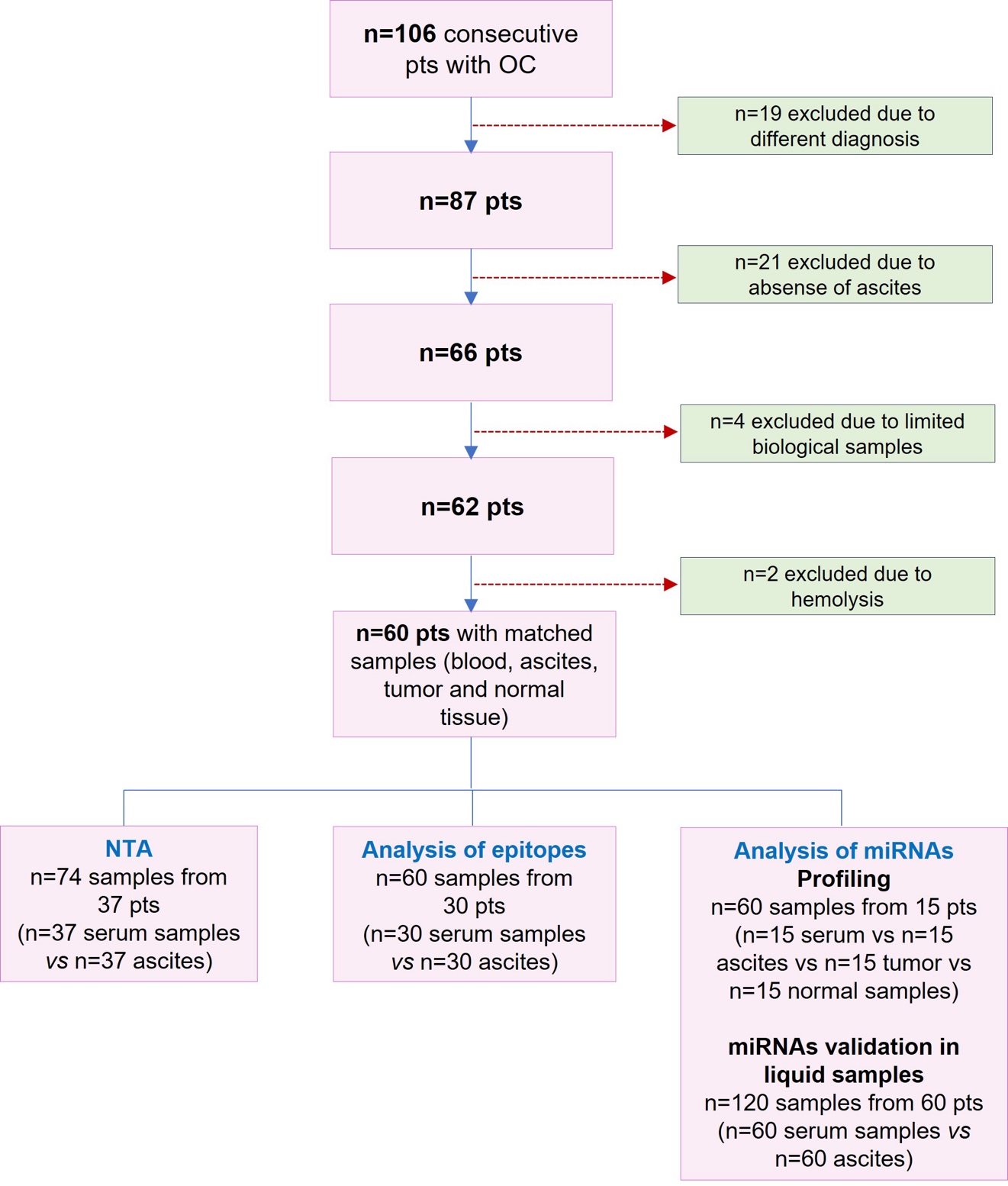


**Supplementary Figure S2: Principal Component Analysis (PCA) showing miRNAs expression in tumor and normal tissues.**


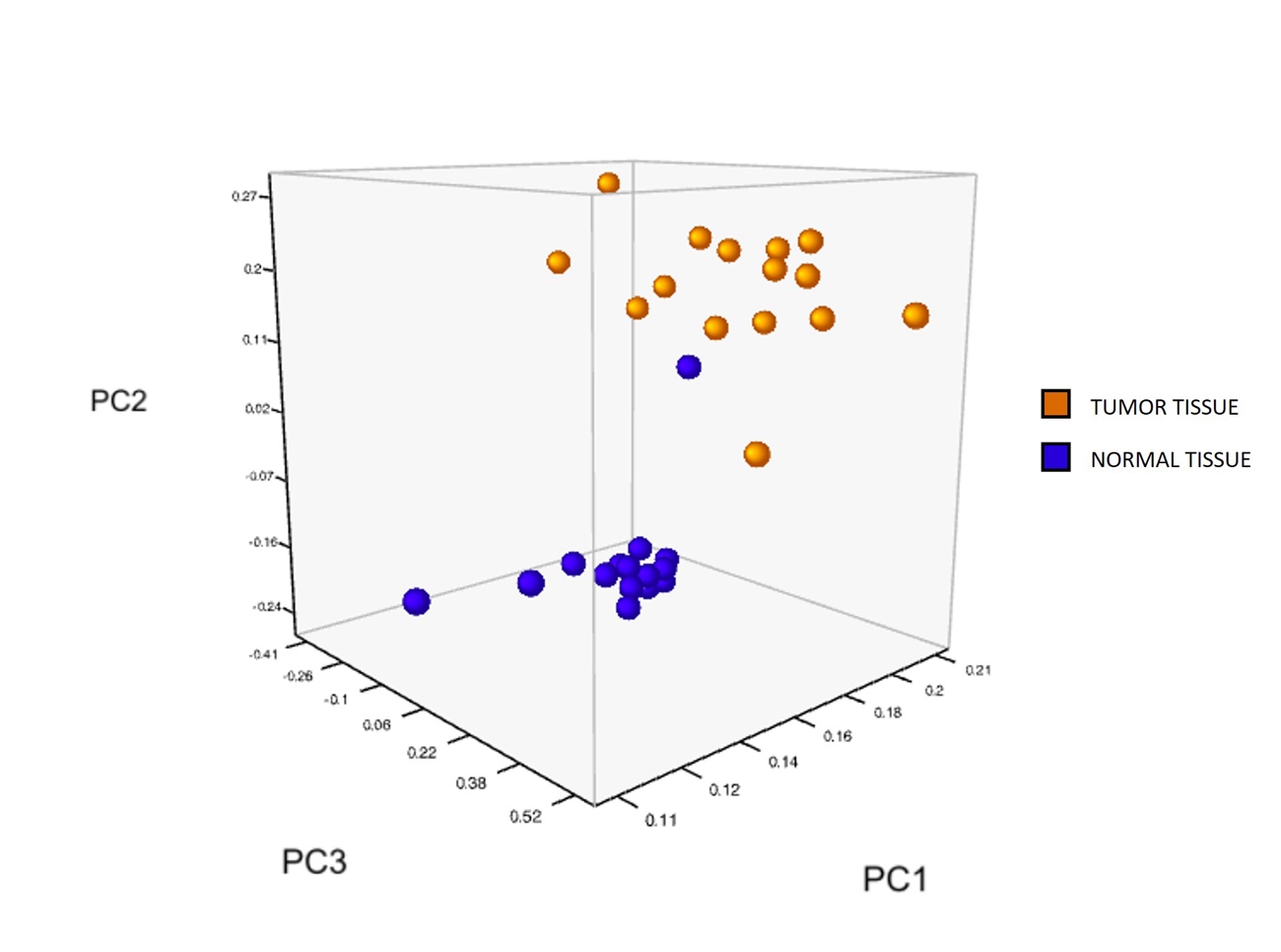


**Supplementary Figure S3: Evaluation of miRNA levels after mimic transfection**


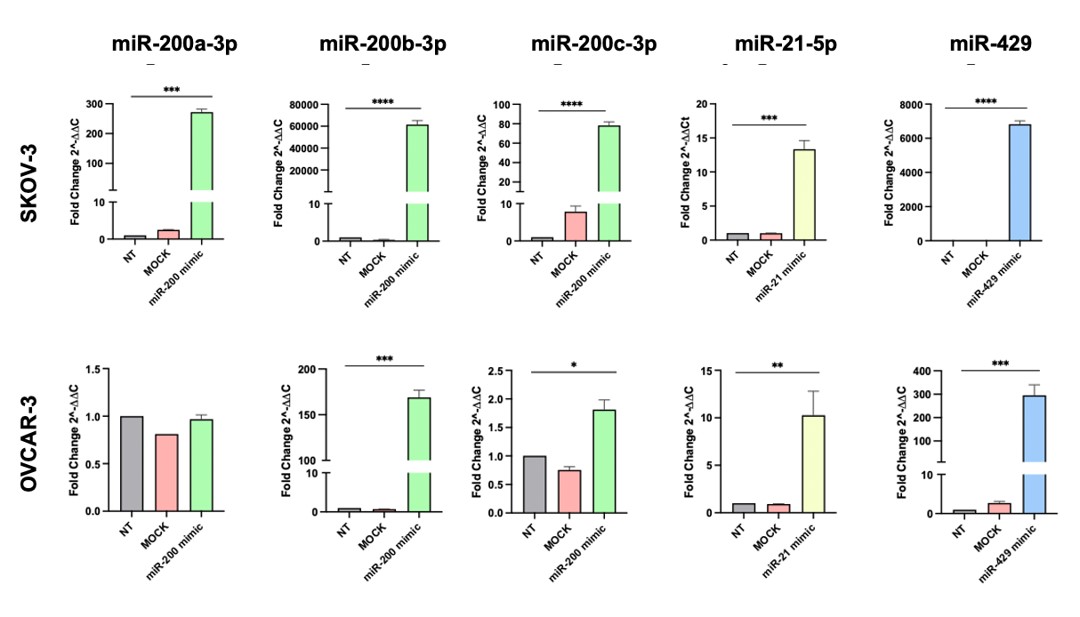

Supplement: Supplementary file 1 — Supplementary material 1. [file 10020_2025_1177_MOESM1_ESM.docx]
